# Development and Characterization of Pharmaceutical Systems Containing Rifampicin

**DOI:** 10.3390/pharmaceutics15010198

**Published:** 2023-01-05

**Authors:** Antonella V. Dan Córdoba, Virginia Aiassa, Jesica A. Dimmer, Camila N. Barrionuevo, Mario A. Quevedo, Marcela R. Longhi, Ariana Zoppi

**Affiliations:** 1 Unidad de Investigación y Desarrollo en Tecnología Farmacéutica (UNITEFA-CONICET), Facultad Ciencias Químicas, Departamento de Ciencias Farmacéuticas, Universidad Nacional de Córdoba, Córdoba 5000, Argentina; 2 Centro de Investigaciones y Transferencia de Villa María (CIT-VM), Universidad de Villa María, Villa María, Córdoba 5900, Argentina; 3 Centro de Estudios e Investigación de la Enfermedad de Chagas y Leishmaniasis, Facultad Ciencias Médicas, Universidad Nacional de Córdoba, Córdoba 5000, Argentina; 4 Instituto de Investigaciones en Ciencias de la Salud, INICSA-CONICET, Córdoba 5000, Argentina

**Keywords:** rifampicin, γ-cyclodextrin, arginine, solubility, stability, antibiofilm activity, antileishmanial activity

## Abstract

Rifampicin is a potent antimicrobial drug with some suboptimal properties, such as poor stability, low solubility, and variable bioavailability. Therefore, in the current study, a multicomponent complex between rifampicin, γ-cyclodextrin, and arginine was prepared with the aim of improving drug properties. Solubility was evaluated by phase-solubility studies. The mechanism of interaction was established through proton nuclear magnetic resonance spectroscopy and molecular modeling. Physicochemical characterization was investigated using Fourier transform-infrared spectroscopy, powder X-ray diffraction, and scanning electron microscopy. The dissolution properties, antimicrobial activity (antibacterial, antibiofilm, and antileishmanial), and stability of the different samples were studied. The results obtained in this investigation demonstrate that multicomponent complexes can improve the water solubility and dissolution rate of rifampicin, as well as its antibacterial and antileishmanial action, and present suitable stability. In conclusion, rifampicin complexed with γ-cyclodextrin and arginine is an attractive approach for developing pharmaceutical dosage forms of rifampicin with increased antimicrobial activities.

## 1. Introduction

The continuous expansion of the global population, along with its greater interconnection, raise the risks that infectious illnesses represent to human health globally [1]. Added to that, incremental increases in antimicrobial resistance is another serious public health problem that imposes a significant social and economic impact [2]. Because infectious illnesses are so prevalent, there is an urgent need to find novel treatment options to address them. In this regard, we have developed an innovative method for preparing drug delivery systems by combining an antimicrobial drug with cyclodextrins (CDs) and amino acids, which improved antimicrobial activity [3,4,5,6,7]. CDs are naturally available cyclic oligosaccharides composed of 6-(α-CD), 7-(β-CD) or 8-(γ-CD) (α-1,4)-linked D-glucopyranose units, arranged in a truncated cone-shaped structure. This conformation allows the formation of binary inclusion complexes (BC), which can modify properties such as solubility, stability, and bioavailability of the included molecule [8,9]. The incorporation of a third compound, such as amino acids, can influence the complexation ability of CDs, resulting in the formation of multicomponent complexes (MC) [10].

Rifampicin (RIF) is an antimicrobial agent that is effective in the treatment of leishmaniasis [11]. This infectious disease is caused by protozoan parasites of the *Leishmania* species, with symptoms ranging from cutaneous lesions to effects on internal organs [12]. Additionally, RIF is employed to treat bacterial infections, with the potential to attack *Staphylococcus aureus* biofilms [13]. Despite its capacity to treat a variety of infectious diseases, the therapeutic efficacy of RIF is currently hampered by its low stability, poor solubility, and irregular bioavailability.

The possibility of using several CDs to prepare RIF inclusion complexes has already been discussed in the literature [14,15]. According to our previous research, complexation with β-CD and arginine (ARG) improves RIF solubility and antimicrobial activity against *S. aureus* biofilms [16]. Since γ-CD has a higher water solubility and a larger central cavity than β-CD, in this work we investigated the capability of γ-CD to form a better MC with RIF and ARG in order to improve its pharmaceutical performance. Furthermore, we postulate that RIF:γ-CD:ARG MC could enhance the antileishmanial activity of RIF. In this regard, solution and solid state MCs were prepared, thoroughly studied, and compared with RIF:ARG and RIF:γ-CD. Phase solubility studies were used to investigate the influence of complexation on drug solubility. To further describe the MC at the atomic level, molecular docking, molecular dynamics, and free-energy binding investigations were combined with NMR measurements. Fourier transform infrared spectroscopy, powder X-ray diffraction, and scanning electron microscopy were used to characterize the MC in solid state. Antimicrobial activity was further tested using a methicillin resistant *S. aureus* (MRSA) biofilm producer bacterial strain and *Leishmania amazonensis* promastigotes. The chemical stability of the MC was also evaluated.

## 2. Materials and Methods

### 2.1. Materials 

RIF was supplied by Parafarm, Buenos Aires, Argentina (CAS: 13292-46-1, 99% pure). γ-CD CAVAMAX W8 (CAS:17465-86-0, 99% pure) was produced by Wacker Chemie (Burghausen, Germany) and donated by Ashland Argentina S.R.L. L-Arginine (CAS: 74-79-3, 98% pure). D_2_O (CAS: 7789-20-0, 99.9 atom% D), ({2,3-bis (2-metoxi-4-nitro-5-sulfofenil)-5-[(fenilamino) carbonilo]-2H-tetrazolium hydroxide (XTT, CAS:111072-31-2, purity ≥ 90%) and trypan blue (CAS:72-57-1) were obtained from Sigma Aldrich^®^ (St. Louis, MO, USA). Mueller Hinton broth (MHB), Mueller Hinton agar (MHA), tryptic soy agar (TSA) and tryptic soy broth (TSB) were provided by Britania (Buenos Aires, Argentina). The water acquired from a Millipore^®^ (Millipore, Bedford, MA, USA)Milli-Q purification system was used in all experiments. Methanol was obtained from Sintorgan^®^ (Buenos Aires, Argentina) and acetonitrile was purchased from J.T.Baker^®^ (Buenos Aires, Argentina).

### 2.2. Phase Solubility Studies 

Phase solubility studies (PSS) are one of the most common techniques to determine the effect of CD and auxiliary substances on drug solubility. According to the technique [17], solubility was evaluated for binary and multicomponent systems. To prepare RIF:γ-CD and RIF:γ-CD:ARG, γ-CD (0–25 mM), and γ-CD:ARG (γ-CD = 0–25 mM and ARG = 5 mM) solutions were added to an excess of RIF (40 mg), respectively. In order to promote dissolution equilibrium, the resulting suspensions were placed in a water bath for 72 h at 25.0 ± 0.5 °C and vortexed twice a day for 15 s. After reaching equilibrium, the suspensions were filtered through a 0.45 µm membrane filter and RIF was quantified by UV spectrophotometer method (Agilent Cary 50 spectrophotometer, Santa Clara, CA, USA) at 334 nm. The determinations were done in triplicate. The stability constants (K_C_) were calculated from the phase solubility profiles, using the slope and the saturation solubility (S_0_) of RIF in the aqueous complexation media (Equation (1)):

(1) $$K_{c\;}=\frac{\mathrm{slope}}{S_{o\;}\left(1-\mathrm{slope}\right)}$$

The complexation efficiency (CE) values were calculated using Equation (2):

(2) $$\mathrm{CE}=\mathrm{slope}\;*\;\left(1-\mathrm{slope}\right)$$

### 2.3. Molecular Modeling 

#### 2.3.1. Modeling of Compounds

The structure of γ-CD was retrieved from the Cambridge Structural Database (CSD) [18], which was deposited under the code CIWMIE10. In order to model this structure, atomic charges and parameters corresponding to the GLYCAM06 force field were assigned [19]. The structures of the RIF and ARG were constructed using the MarvinSketch software kindly provided by ChemAxon [20], with their ionization states being modeled at pH 6.8. Both compounds were assigned charges and molecular parameters corresponding to the GAFF2 forcefield [21].

#### 2.3.2. Molecular Docking Studies

Molecular docking procedures were performed using the AutoDock-GPU docking engine [22], with affinity grids being calculated using the AutoGrid4 tool [23]. Affinity grids were constructed using a cubic box (80 Å × 80 Å × 80 Å) placed using as reference the center of mass of the host molecule. As stated previously, atomic charges used to calculate affinity grids corresponded to the GLYCAM06 force field. Docking runs were performed using a Lamarckian Genetic Algorithm combined with a Local search approach (GA-LS). The lowest energy docked conformation was considered as the resulting docked structure, with the docking assay being performed 100 times in order to evaluate alternative and/or consistent binding modes. The energetic profile of the interaction between the host and guest molecule was further analyzed by means of the Molecular Mechanics Generalized Born Surface Area (MMGBSA) method, as implemented in the MMPBSA.py script [24].

#### 2.3.3. Molecular Dynamics Simulations

Trajectories corresponding to the molecular dynamics studies were obtained using the Amber22 software package [25]. Atomic charges and molecular parameters corresponding to guest molecules (RIF and ARG) were assigned from the GAFF2 force field [21], while the host molecule was modeled using the GLYCAM06 force field [19]. Trajectories were obtained using a starting structure that resulted from the molecular docking experiments, which were solvated with an SPCE preequilibrated water model [26]. Multiple simulation stages were performed, including two minimization (solvent and whole system) phase as well as a heating (0 K to target temperature, 5 ns), equilibration (10 ns) and production phase (100 ns).The SHAKE algorithm was applied to constrain all bonds involving hydrogen atoms, with a simulation time step of 2 fs. Analyses of the MD trajectories were performed using the MDAnalysis python library [27], and visualized using VMD v.1.9 [28]. Trajectories were obtained from Compute Unified Device Architecture-designed code (pmemd.cuda), with computational facilities provided by the Centro de Computación de Alto Desempeño (CCAD) [29], Universidad Nacional de Córdoba, Argentina.

#### 2.3.4. Selection of Target Temperature for MD Simulations

Taking into account that experiments were carried out at a fixed temperature of 25 °C (298 K), and in order to adequately model the temperature effect during the MD studies, the simulation of γ-CD within a solvent box was studied at 290, 293, 295 and 298 K. From the resulting simulations, the temperature range of the system was analyzed. In order to assure the efficacy of the applied thermostat algorithm, the target temperature for simulations was selected in such a way that the temperature did not exceed 298 K in 15% of the simulated trajectory. The resulting temperature profiles are shown in Figures S25–S28. Based on those simulations, MD trajectories were obtained with a target temperature of 293 K.

### 2.4. Proton Nuclear Magnetic Resonance Spectroscopy

In order to examine how RIF, γ-CD, and ARG interact among themselves, samples were dissolved in D_2_O and analyzed using a Bruker Avance II spectrometer at 298 K. As an internal reference, the resonance caused by the remaining solvent (HDO) at 4.8 ppm was employed. Then, ^1^H-NMR spectra were obtained for pure components, BC (RIF:ARG and RIF:γ-CD) and MC (RIF:γ-CD:ARG), and the change in the chemical shifts (Δδ) was calculated using the following Equation (3):

(3) $$∆\delta =\delta_{\mathrm{complex}}-\delta_{\mathrm{free}}$$

### 2.5. Preparation of Inclusion Complexes in Solid State

Solid systems were prepared by two methods: kneading (KN) and freeze-drying (FD) methods. The complexes were also obtained by physical mixing (PM) as a control. In all cases, systems were prepared at a 1:1 and 1:1:1 molar ratio for BC and MC, respectively.

Physical mixing: the components corresponding to each system were weighed and added to a mortar. The samples were mixed for 5 min without the addition of any solvent.

Kneading method: the solid components were placed in a mortar. A specific volume of H_2_O was added according to the amount of total solid (0.25 µL of H_2_O per mg of solid) and mixed for 30 min.

Freeze-drying: the systems were prepared in solution. The resulting pH was adjusted to 6.8 by the addition of NaOH (0.1 M). The solutions were stored at −40 °C prior to freeze-drying using a Freeze Dry 4.5 Labconco Corp., Kansas City, MI, USA.

### 2.6. Fourier Transform-Infrared Spectroscopy and Powder X-ray Diffraction 

The FTIR studies were achieved to evaluate the interaction between RIF, γ-CD, and ARG in complexes prepared by PM, KN, and FD techniques. The FTIR spectra were obtained from an Agilent Cary 630 FTIR spectrometer with a range of 4000–500 cm^−1^. A PANanalytical X’Pert PRO diffractometer with Cu-Kα radiation was used to assess changes in sample crystallinity. The PXRD patterns were obtained by employing a diffraction angle (2θ) from 4 to 35° with a step size of 0.026° and a scanning rate of 23.0 s step ^−1^.

### 2.7. Scanning Electron Microscopy

The morphological structures were investigated using a Carl Zeiss Sigma Scanning Electron Microscopy (SEM, Zeiss, Jena, Germany) at the Laboratorio de Microscopía y Análisis por Rayos X (LAMARX) of the Universidad Nacional de Córdoba, Argentina. The samples were fixed to a brass stub using double-sided aluminum tape. They were gold-palladium coated under vacuum using a sputter coater (Quorum 150) in order to improve the conductivity of samples.

### 2.8. Dissolution Studies

The dissolution studies were carried out to evaluate the effect of inclusion complex formation on RIF release. The paddle method (USP apparatus 2) was used in the experiments, which were carried out using a Hanson SRII 6 Flask Dissolution Test Station (Hanson Research Corporation, Chatsworth, CA, USA). Pure RIF (50 mg) and its equivalent quantity in the systems (RIF:ARG, RIF:γ-CD, and RIF:γ-CD:ARG) were submerged in 300 mL of simulated intestinal fluid at pH 6.8 at 37.0 ± 0.5 °C with stirring at 50 rpm. At different time intervals, aliquots of the sample (2 mL) were withdrawn and replaced with fresh dissolution medium to maintain sink conditions. Then, the samples were filtered using a 0.45 µm membrane filter (Millipore, Burlington, MA, USA) and measured by a UV-Vis spectrophotometer (Agilent Cary 50 spectrophotometer) at 334 nm. For comparing the dissolution profiles, an independent-model method was used, and the similarity factor *f_2_* values were calculated (Equation (3)):

(4) $$f_{2}=50\log\left\{{\left[1+\frac{1}{n}\overset{R}{\underset{t-1}{\sum }}{\left(R_{t}-T_{t}\right)}^{2}\right]}^{-0.5}\right\}\times 100$$

where *n* is the number of time points, *R_t_* is the percentage of the reference sample (pure RIF) that has been dissolved at each time point, and *T_t_* is the percentage of the test sample (inclusion complexes) that has been dissolved at each time point. When the *f*_2_ value was equal to or greater than 50, it was determined that the profiles were statistically similar.

### 2.9. Microbiological Studies

#### 2.9.1. Bacterial Strain and Growth Conditions

A methicillin-resistant clinical isolate of *S. aureus* 771 (MRSA 771) generously donated by Sanatorio Aconcagua in Córdoba, Argentina, was used in this study. Glycerol 10% (*v*/*v*) was used to preserve stock cultures at a temperature of −70 °C. TSA was used to cultivate the strains. A bacterial culture was prepared by inoculating one colony in TSB.

#### 2.9.2. Determination of the Minimum Inhibitory Concentration of RIF

According to the Clinical Laboratory Standards Institute (CLSI) [30], the minimum inhibitory concentration (MIC) was determined using the standard tube dilution method. The bacterial strain cultured for 24 h in MHA was diluted to 1 × 10^6^ colony-forming units per milliliter (CFU/mL) using MHB. The inoculum was incubated for 10 min at 35.0 ± 0.5 °C. Then, RIF was added at different concentrations (0.0075–8.000 µg/mL). Bacterial growth was assessed visually according to CLSI guidelines after 18 h of incubation. MIC was determined as the lowest concentration of RIF at which no turbidity was observed. Bacteria controls, without RIF, were run in parallel.

#### 2.9.3. In Vitro Antimicrobial Study

Pure RIF, ARG, γ-CD, BC (RIF:ARG and RIF:γ-CD) and MC (RIF:γ-CD:ARG) were evaluated for in vitro antimicrobial studies. For this study, MHA plates were swabbed with MRSA 771 (Inoculum ~0.5 Mc Farland). Then, a 6 mm-diameter disc of sterile Whatman no. 1 membrane filters containing the pure drug, or BC and MC (RIF 5 µg/mL), was placed resting on the agar plates. After a diffusion period of 30 min, the plates were kept for incubation at 37 °C for 18 h, and the zone of inhibition was determined in triplicate.

#### 2.9.4. Evaluation of the Metabolic Activity of the Biofilm

##### Biofilm Formation and Treatment

In order to evaluate the biofilm formation capacity and study the effect of ARG and γ-CD against MRSA biofilm, microbiological studies were performed. Biofilms were formed in 96-well plates containing 200 μL of MRSA 771 suspension (1 × 10^6^ CFU/mL) in TSB, supplemented with 0.25% glucose to promote the formation of biofilms. Plates were incubated for 24 h at 35.0 ± 0.5 °C on an orbital shaker (130 rpm). After 24 h, the planktonic cells were carefully removed, and the biofilm was washed twice with 200 μL of phosphate buffer saline (PBS). Then, a fresh nutrient medium containing pure ARG and γ-CD with the same concentrations that would be used in the BC and MC systems was added to the biofilms. Plates were further incubated for 24 h at 35.0 ± 0.5 °C. Finally, after 24 h of exposure to pure ARG and γ-CD, XTT assays were performed. The evaluation of the effect of RIF on determining the metabolic activity of the biofilm by means of the XTT assay was not carried out since RIF absorbs at the same wavelength as the reduced XTT.

##### XTT Assay

The XTT reduction assay was used for the quantification of the metabolic activity of MRSA 771 cells in the biofilm. After exposure to pure ARG and γ-CD at the same concentrations that would be used in the BC and MC systems, the biofilms were washed with PBS. Next, 250 μL of a solution containing 20 µg/L of phenazine methosulfate and 200 µg/L of XTT was added to each well. The microtiter plates were incubated in the dark at 35.0 ± 0.5 °C for 3 h. The absorbance was determined in a Biotek Synergy HT multimode microplate reader at 490 nm.

#### 2.9.5. Biofilm Analysis by Scanning Electron Microscopy

##### Biofilm Preparation

For the purpose of studying the effect of RIF complexation on its antibiofilm activity, MRSA 771 biofilms were formed. Coverslips were placed into suspensions of MRSA 771 (1 × 10^6^ CFU/mL) in TSB supplemented with 0.25% glucose. Then, the suspensions were incubated for 24 h at 35.0 ± 0.5 °C on an orbital shaker (130 rpm) to promote biofilm formation. Next, the slides were washed with 2 mL of PBS for 10 s to remove non-adherent bacteria. The biofilms formed were introduced into a fresh nutrient medium containing pure RIF, BC (RIF:ARG and RIF:γ-CD) and MC (RIF:γ-CD:ARG) at the same concentration of RIF (4 mg/mL). As a control, the biofilm without treatment was used. Subsequently, the samples were incubated for 24 h at 35.0 ± 0.5 °C.

##### Sample Preparation for SEM

For this analysis, coverslips were washed with 2 mL of PBS for 10 s. Then, the biofilms were dehydrated using a series of solutions with increasing concentrations of ethanol (30%, 50%, 70%, 80%, 90% and pure ethanol) for 10 min each. Finally, coverslips were placed on slides and plated with gold/palladium using a Quorum 150 metallizer. SEM analyses were performed in a Carl Zeiss Sigma Scanning Electron Microscopy (SEM) at the Laboratorio de Microscopía y Análisis por Rayos X (LAMARX) of the Universidad Nacional de Córdoba, Argentina.

### 2.10. Antileishmanial Activity

#### 2.10.1. Promastigotes Culture

*L. amazonensis* (MHOM/VE/84/MEL) were kindly provided by Dr. Esteban Lozano (Instituto de Medicina y Biología Experimental de Cuyo, IMBECU-CONICET, Argentina). They were maintained in modified NNN medium with RPMI 1640 medium supplemented with 2 mM L-glutamine, 100 U/mL penicillin, 100 mg/mL streptomycin (all from Sigma) and 10% of heat inactivated fetal bovine serum (FBS, Natocor) at 25 °C. Seven days before assays, they were transferred to Diamond’s liquid medium (0.1 M NaCl, 0.05 M K_2_HPO_4_, 0.625% tryptone, 0.625% tryptose, and 0.625% yeast extract, adjusted to pH 7.2), supplemented with 10% of FBS, hemine 12.5 µg/mL, 100 U/mL penicillin, and 100 mg/mL streptomycin (all from Sigma), and cultured at 25 °C.

#### 2.10.2. Antipromastigote Assay

##### Antipromastigote Assay

First, the IC50 value of pure RIF (concentration that inhibited 50% of parasite growth) was determined. For this purpose, a seriated dilution of RIF ranging from 75 µg/mL to 6.25 µg/mL was incubated with a suspension of 3 × 10^6^ parasites/mL. The same suspension was then incubated in another independent assay with experimental groups (n = 3) divided as follows: RIF (at the IC50), RIF:ARG, RIF:γ-CD, and RIF:γ-CD:ARG prepared to give the same concentration as RIF. Negative controls (NC, promastigotes without treatments), ARG and γ-CD controls (at the same concentrations used in the RIF:ARG and RIF:γ-CD studies) were also included. The trypan blue exclusion test was used to determine the percentage of cell viability (CV) after 72 h of each assay [31]. The results were expressed as a percentage of CV (% CV) with respect to NC (100% CV). Every experiment was performed in triplicate.

### 2.11. Stability Studies

#### 2.11.1. Chromatographic Conditions

Based on a method reported by Liu et al. [32], a stability-indicating HPLC assay method for RIF was developed and validated. Chromatographic separation was performed by using an Agilent S1100 HPLC (Agilent Technologies, Santa Clara, CA, USA) equipped with a G1310A isocratic pump, a G1313A autosampler, a G1365B UV detector, and a G1316A column heater. A C8 column with a size of 250 mm × 4.6 mm (Phenomenex, Torrance, CA, USA) was used as a stationary phase. The mobile phase employed was a mixture of methanol–acetonitrile–monopotassium phosphate (pH 5, 10 mM) (25:35:40 *v*/*v*), with a flow rate of 1.5 min/mL at 35 °C. The injection volume was 20 µL, and the detection wavelength was 254 nm. 

#### 2.11.2. Degradation Studies

In order to prove the specificity of the HPLC method, forced degradation studies were carried out. RIF solutions (5 mg/mL) were subjected to acidic (1 M HCl), basic (1 M NaOH), and oxidative (30% H_2_O_2_) stress conditions. Then, solutions exposed to HCl and NaOH were neutralized (with 1 M NaOH or 1 M HCl, respectively). After degradation, solutions were diluted in the mobile phase and filtered through a 0.45 μm filter before injection.

#### 2.11.3. Validation Method

The validation of the HPLC method was performed by determining the parameters of linearity, accuracy, precision, detection limit (DL), and quantification limit (QL), according to the ICH Q2 (R1) guidelines [33].

##### Linearity

To determine the linearity of the analytical procedure, 25 mg of RIF wasweighed in 25 mL of mobile phase. The concentration ranges studied were 50, 80, 100, 120, and 150 μg/mL. The solutions were filtered and quantified. The experiment was performed in triplicate.

##### Accuracy

The accuracy was studied by the recovery determination of RIF solutions at concentrations of 50, 100, and 150 µg/mL. The accepted reference value was 98–102% recovery.

##### Precision

The precision of the analytical procedure was evaluated by determining the repeatability and intermediate precision parameters. The RIF concentrations were 50, 100 and 150 µg/mL. Repeatability was evaluated under the same conditions and on the same day. Intermediate precision was determined by performing inter-day tests. The analysis of the results was carried out by calculating the coefficient of variation (*CV*) (Equation (5)):

(5) $$CV\;\left(\%\right)=\frac{SD}{x}*\;100\;\;$$

where *SD* is the standard deviation, and *x* is the average value of the measurements. Values less than 2% and 3% were considered acceptable for the repeatability and intermediate precision parameters, respectively.

##### Detection and Quantification Limits

The *DL* and *QL* values were obtained from the RIF calibration curve. The calculation was performed as mentioned in Equations (5) and (6):

(6) DL=3.3 σ/S

(7) QL=10 σ/S

where *σ* is the standard deviation of the response, and *S* is the slope of the RIF calibration curve.

##### Stability Evaluation

The chemical stability of RIF pure and in the systems (RIF:ARG, RIF:γ-CD, and RIF:γ-CD:ARG) was determined at 70 °C in pH 6.8 phosphate buffer solutions. Solutions were prepared by dissolving 20 mg of RIF in 100 mL of the aqueous buffer containing equimolar concentrations of ARG, γ-CD, and γ-CD:ARG, which were then placed in a thermostated water bath (Circulators HAAKE F3-K, Germany). Samples were taken at various time intervals. Solutions were diluted in the mobile phase and filtered through a 0.45 μm filter before injection. The quantification of RIF was carried out by applying the HPLC method mentioned in Section 2.11.1.

## 3. Results and Discussion

### 3.1. Phase Solubility Studies 

The phase solubility diagrams of RIF with γ-CD in the absence or presence of ARG demonstrated a linear increase in RIF solubility and can be classified as A_L_ type, indicating a 1:1 complex formation (slope less than 1). The corresponding stability constant and CE values calculated from the phase-solubility diagrams were 92 ± 2 M^−1^ and 0.101, and 105 ± 5 M^−1^ and 0.126 for RIF:γ-CD and RIF:γ-CD:ARG, respectively. The solubility of pure RIF at pH 6.8 was 1.27 ± 0.02 mg/mL, while in γ-CD solution the solubility of the drug was 3.61 ± 0.05 mg/mL and in the presence of γ-CD and ARG solution, it was 4.20 ± 0.03 mg/mL. The RIF solubility increment and stability constant observed for RIF:γ-CD:ARG was greater than that observed for RIF:β-CD:ARG MC (2.10 ± 0.01 mg/mL and 61 ± 2 M^−1^) [16]. These findings show that the presence of ARG improves the solubilizing ability of γ-CD and point to the formation of an MC between these compounds. 

### 3.2. Molecular Modeling of Binary Complexes

#### 3.2.1. Molecular Modeling of Binary Complexes

**Figure 1:**
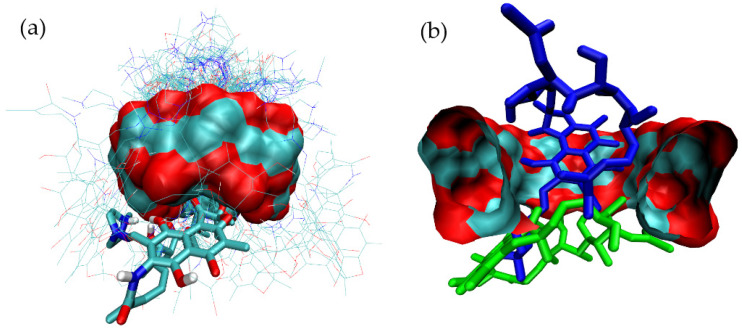
Results corresponding to the lowest energy binding modes for 100 independent docking runs of RIF to γ-CD. (**a**) Overall results of docking results for the binding of the RIF:γ-CD complex and (**b**) Selected results of docking results for the binding of the RIF:γ-CD complex.

In order to predict the formation of the corresponding BC, RIF was docked to γ-CD. The RIF molecule was modeled in its zwitterionic state as expected for the experimental pH conditions. Results corresponding to the 100 independent docking runs of RIF are shown in Figure 1.

**Figure 2:**
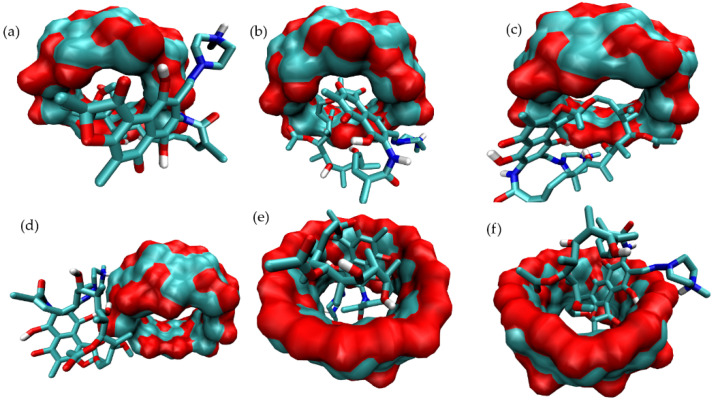
Three-dimensional views of the different binding modes clusters corresponding to the binding of RIF to γ-CD as obtained after 100 independent docking runs. (**a**) mode-1, (**b**) mode-2, (**c**) mode-3, (**d**) mode-4, (**e**) mode-5 and (**f**) mode-6.

After structural analysis considering the symmetry of γ-CD with respect to its central axis, six main binding modes for RIF were identified (Figure 2a–f).

Figure 2a–d presents the main structures of the complexes in which the inclusion of part of RIF within the internal cavity of γ-CD occurs facing from the narrow rim of the host molecule. In contrast, inclusion modes shown in Figure 2e,f corresponds to the interaction of RIF with the external surface of γ-CD. In order to identify the most stable binding mode, these reference complexes were subjected to MD simulations at the target temperature, with the corresponding free-energy binding analysis being performed to assess the stability of the complex in each case. Table 1 presents the results obtained for the stability of the complexes after applying the corresponding free-energy analysis by means of the MMPBSA methodology.

**Figure 3:**
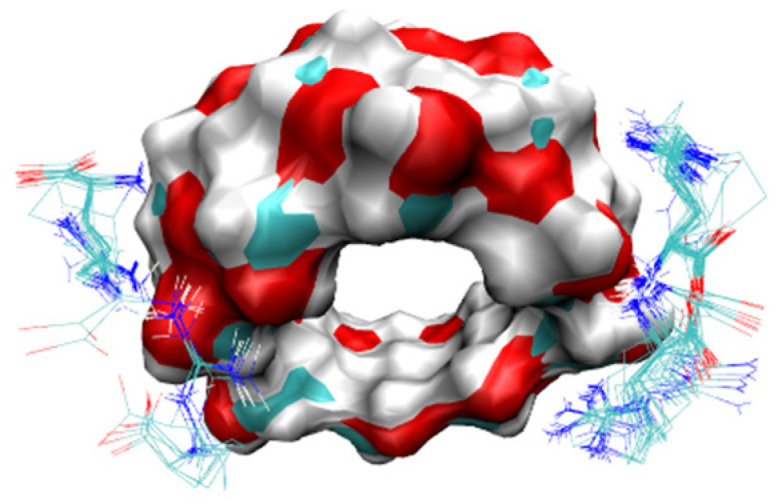
Results corresponding to the lowest energy binding modes for 100 independent docking runs of ARG to γ-CD.

In all cases, a balance between the VDW and ELE components is observed as the driving force for the binding. As is shown in the table, two particular binding modes (i.e., mode-3 and mode-5) represent the structures of interest for further analysis of the MC structure. Figures S2 and S3 show the distance between the centers of mass of the guest and host molecules for these two binding modes, evidencing that both inclusion complexes are maintained throughout the MD simulation. An homologous analysis was performed to study the nature of the formation of a BC between ARG and γ-CD. Figure 3 presents the overall results obtained for the 100 docking runs.

**Figure 4:**
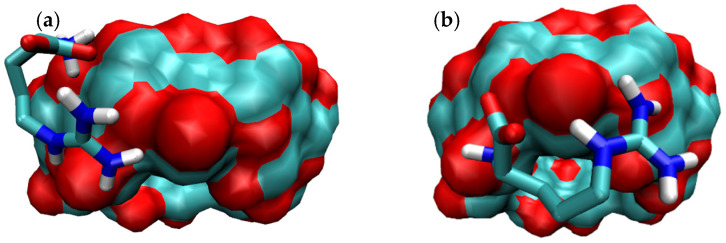
Main binding modes obtained for the docking of ARG to γ-CD. (**a**) Binding mode-1 for the ARG:γ-CD complex and (**b**) Binding mode-2 for the ARG:γ-CD complex.

In this case, the most stable binding modes found for ARG show two alternative binding modes, both of them including the interaction of the guest molecule with the outer surface of γ-CD. Figure 4 presents these binding modes, which were further subjected to MD simulations in order to assess their thermodynamic stabilities.

The corresponding calculated free-energy components for the interaction of each binding mode are shown in Table 2.

As can be seen, the thermodynamic stability of the binding of ARG to γ-CD is considerably lower than that of RIF. Moreover, as presented in Figures S4 and S5, the intermolecular interaction between ARG and γ-CD is not maintained throughout the MD trajectory. Taking into account the discussed findings, both mode-3 and mode-5 were further subjected to studies regarding their ability to form MC with ARG.

#### 3.2.2. Molecular Modeling of Multicomponent Complexes

As was commented above, the modeling of the RIF:γ-CD:ARG complex was initiated from the most stable BC mode-3 and mode-5. In order to consider the dynamic nature of the BC, MD simulations were performed for both modes, and afterwards structural clusters were identified by analyzing the RMSD throughout the trajectory. As done for the BC, the structure of the MC was studied by iteratively docking the amino acid to each representative structure, obtaining a full set of docked poses corresponding to 100 experiments.

**Figure 5:**
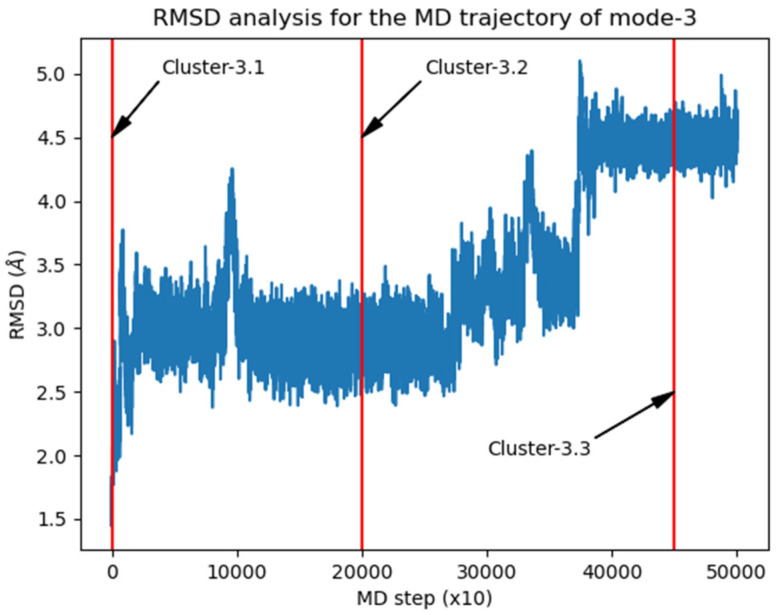
RMSD vs. frame plot for the MD trajectory corresponding to the binary complex mode-3.

The RMSD analysis over the MD trajectory corresponding to the binary complex mode-2 is presented in Figure 5. Three main complex conformations were identified, constituting the initial structures for the exploration of the corresponding ternary complex.

**Figure 6:**
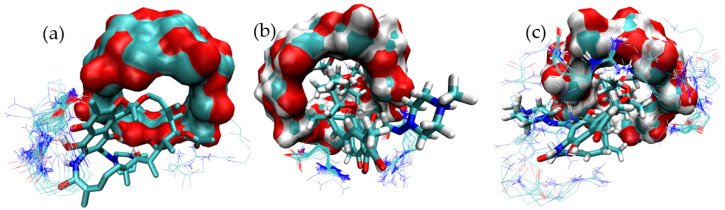
Binding modes identified for the MC formed by RIF:γ-CD:ARG from structures clustered from mode-3. Docking results for the binding of ARG to mode-3.1 (**a**), mode-3.2 (**b**) and mode-3.3 (**c**) of the RIF:γ-CD BC.

Figure 6 presents the full set of docked structures obtained for the ternary complex starting from each of the selected structures corresponding to the binary complex mode-3.

**Figure 7:**
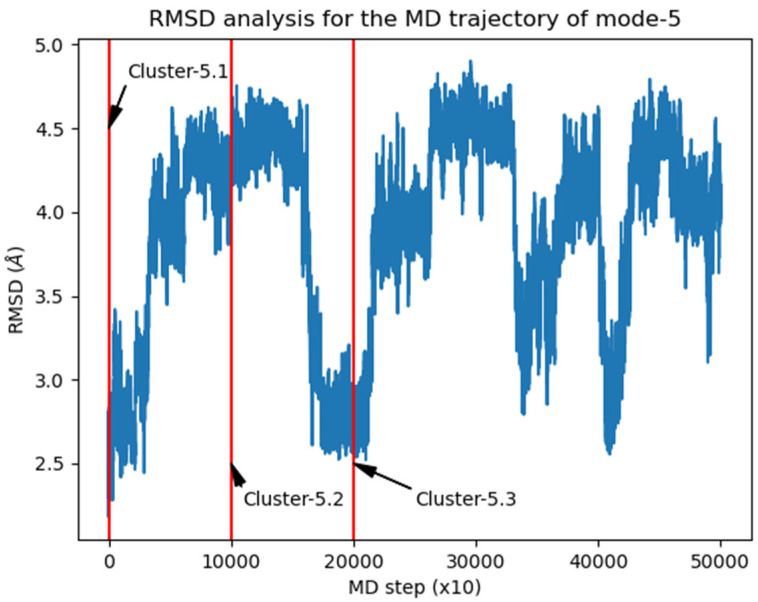
RMSD vs. frame plot for the MD trajectory corresponding to the BC mode-5.

**Figure 8:**
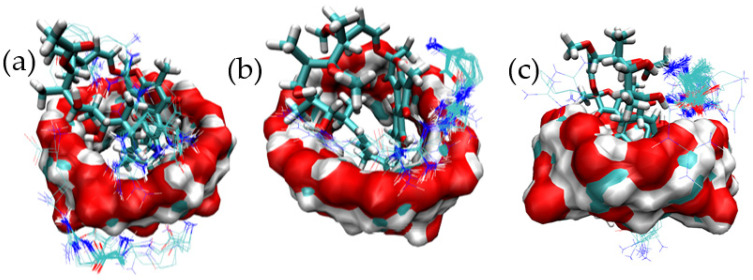
Binding modes identified for the MC formed by ARG:RIF:γ-CD from structures clustered from mode-5. Docking results for the binding of ARG to mode-5.1 (**a**), mode-5.2 (**b**) and mode-5.3 (**c**) of the RIF:γ-CD BC.

The same structural analysis was performed for the binary complex mode-5 (Figure 7). Again three structures were isolated and subjected to further studies in order to predict potential ternary complexes (Figure 8).

The structure of the corresponding ternary complex was predicted by systematically docking ARG to each substructure identified for mode-3 and mode-5. Several binding modes of ARG were identified to each initial structure, and after applying MD simulations, the most stable ternary complex was identified by observing the distance between RIF and ARG, and computing the inclusion of RIF within γ-CD. The corresponding plots are shown in Figures S6–S24. From this structural analysis, it was concluded that the MC corresponding to mode-3.3.4 (Figure S14) was the only one maintaining its structure, with RIF being included within the γ-CD cavity and the amino acid interacting with both molecules (Figure 9).

Table 3 presents the MMPBSA quantification of the energy interaction components calculated for mode-3.3.4. When the inclusion stability of RIF in this MC is compared to that of the corresponding binary complex, a slightly increased stability (i.e., 1.42 kcal/mol) is observed in the former one (−32.89 and −31.47 kcal/mol, for the multicomponent and binary complex, respectively).

#### 3.2.3. Interaction between RIF and ARG 

**Figure 9:**
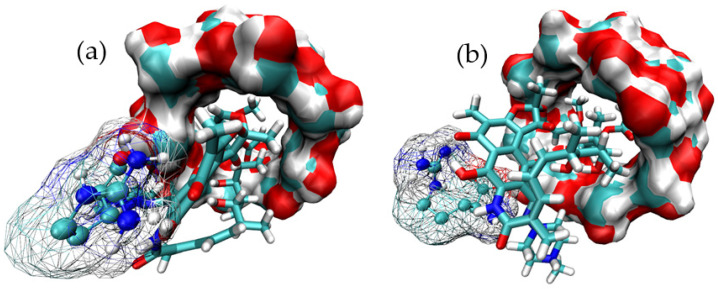
Structure of the most stable MC formed by RIF:γ-CD:ARG. Three-dimensional view of the most stable MC formed between RIF:γ-CD:ARG (mode-3.3.4): (**a**) view A and (**b**) view B.

**Figure 10:**
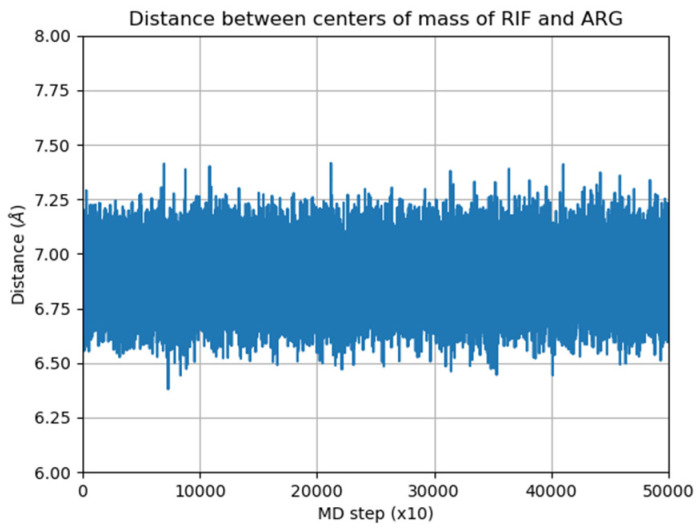
Distance between the centers of mass of RIF and ARG in solution.

In order to study the interaction between RIF and ARG in the absence of γ-CD, the MC shown in Figure 9 was used as the starting structure. In this case, the γ-CD molecule was deleted, and the resulting structure was further subjected to MD simulations under the same conditions as those used before. Figure 10 presents the distance between the centers of mass of both molecules throughout the simulation.

**Figure 11:**
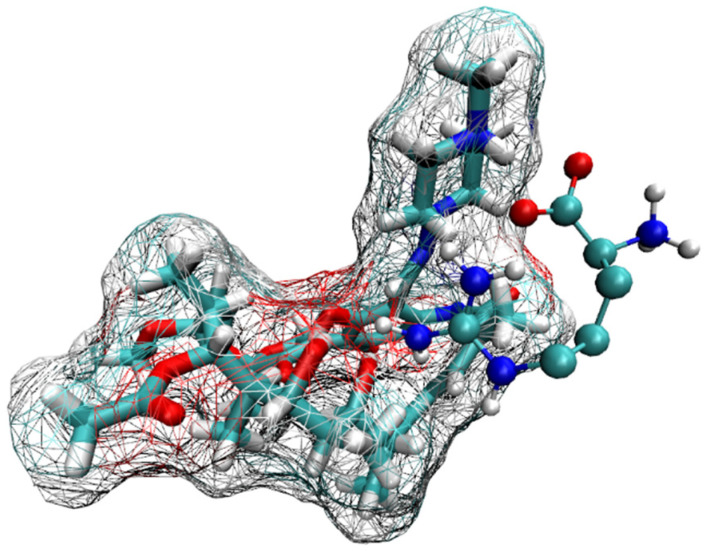
Three-dimensional model of the RIF and ARG interacting in solution.

As can be seen in Figure 11, both ligands are able to maintain intermolecular interactions in solution, throughout the simulated trajectory. Table 4 presents the calculated intermolecular interaction components as determined by applying the MMPBSA method. In this case, the intermolecular association is predominantly governed by electrostatic interactions.

### 3.3. Proton Nuclear Magnetic Resonance Spectroscopy

**Figure 12:**
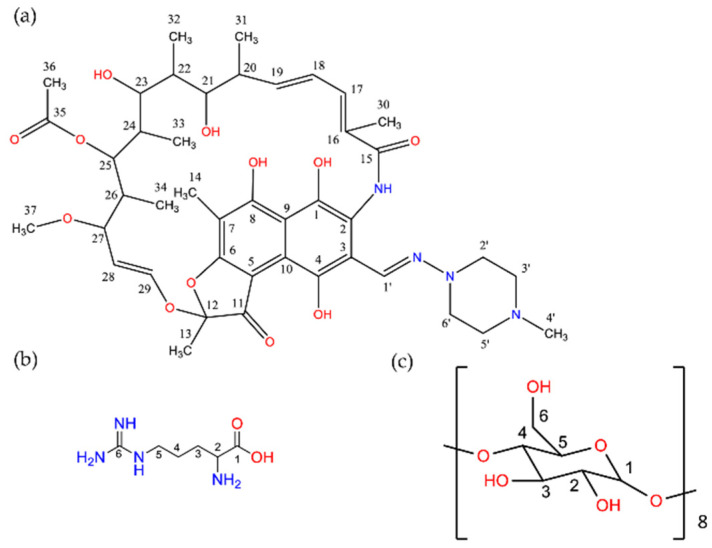
Molecular structures and NMR signal notation of: (**a**) RIF, (**b**) ARG and (**c**) γ-CD.

^1^H NMR experiments were performed to complement molecular modeling studies. The formation of inclusion complexes was evidenced by comparing the chemical shift displacements of RIF, γ-CD, and ARG with their respective BC and MC, as shown in Table 5 (see Figure 12 for proton numbering). For the RIF:γ-CD BC, the major induced shielding was observed for the protons of the internal cavity of γ-CD (H5 and H6). These findings confirmed the inclusion of RIF in the γ-CD cavity. In addition, marked upfield displacement in chemical shifts were observed for the H24, H30, H33 and H36 protons of RIF, implying that this moiety was positioned in a high electronic density environment (glycosidic linkage oxygens of γ-CD that are rich in π electrons), resulting in a shielding effect, as seen in the theoretical studies. In addition, H18, H13, H2′-H6′, and H4′ protons of RIF show displacement in chemical shifts, which could indicate conformational changes of the drug after forming BC. RIF:γ-CD:ARG MC showed remarkable changes in the chemical shifts of the RIF, γ-CD and ARG protons. Significant chemical shifts for both internal and external protons were observed for γ-CD, confirming a deeper inclusion of RIF in the internal cavity and the position of ARG on the outer side surface of the macromolecule, which was consistent with molecular modeling results. In the case of the RIF:ARG system, RIF and ARG proton resonances exhibited both upfield and downfield displacements, demonstrating the presence of intermolecular interactions between the compounds.

### 3.4. Characterization of Systems in the Solid State by Fourier Transform-Infrared Spectroscopy, Powder X-ray Diffraction, Thermal Analysis and Scanning Electron Microscopy

**Figure 13:**
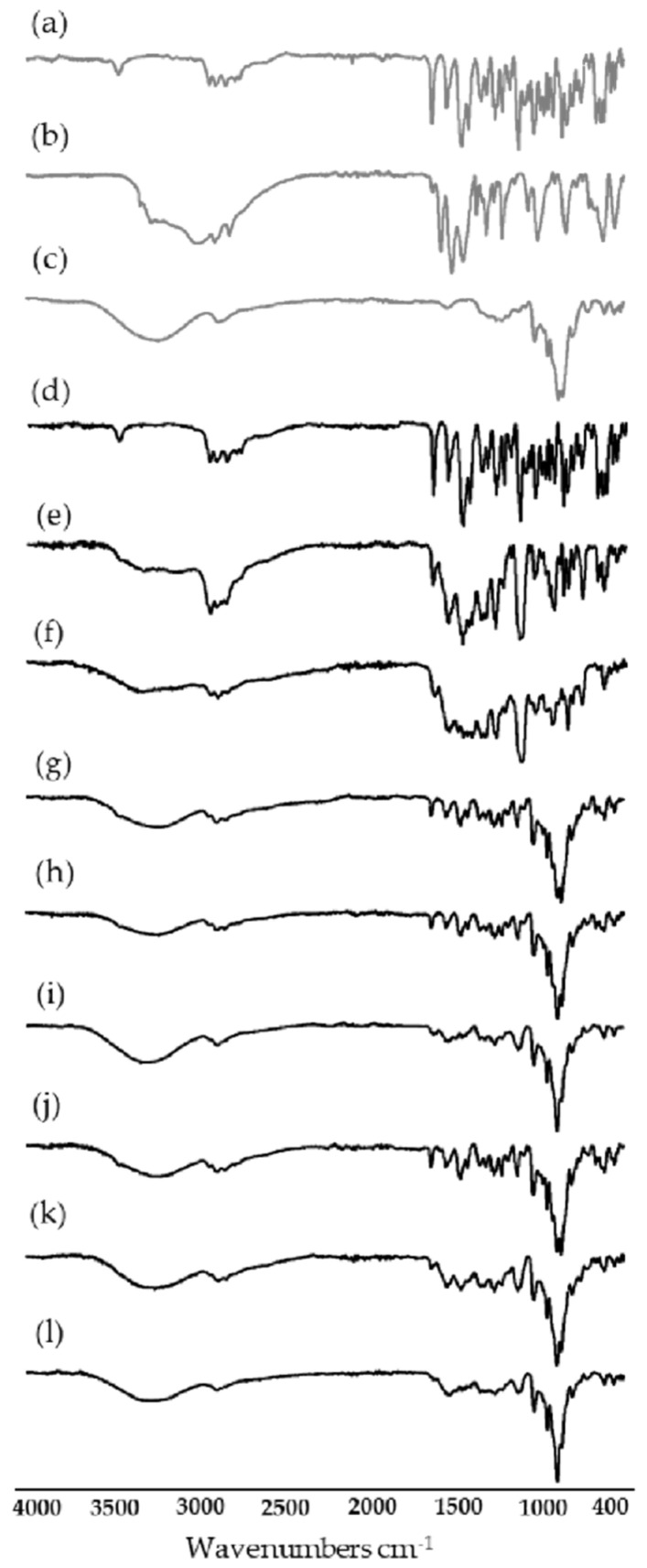
IR spectra of RIF (**a**), ARG (**b**), γ-CD (**c**), RIF:ARG PM (**d**), RIF:ARG KN (**e**), RIF:ARG FD (**f**), RIF:γ-CD PM (**g**), RIF:γ-CD KN (**h**), RIF:γ-CD FD (**i**), RIF:γ-CD:ARG PM (**j**), RIF:γ-CD:ARG KN (**k**), and y RIF:γ-CD:ARG FD (**l**).

**Figure 14:**
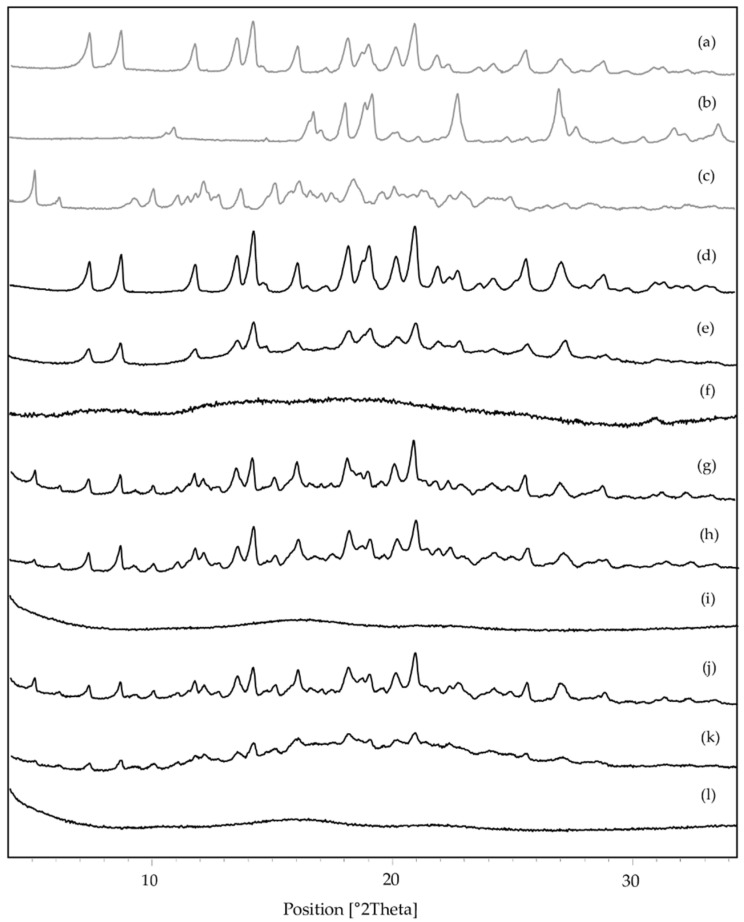
PXRD diffractogram of RIF (**a**), ARG (**b**), γ-CD (**c**), RIF:ARG PM (**d**), RIF:ARG KN (**e**), RIF:ARG FD (**f**), RIF:γ-CD PM (**g**), RIF:γ-CD KN (**h**), RIF:γ-CD FD (**i**), RIF:γ-CD:ARG PM (**j**), RIF:γ-CD:ARG KN (**k**), and RIF:γ-CD:ARG FD (**l**).

**Figure 15:**
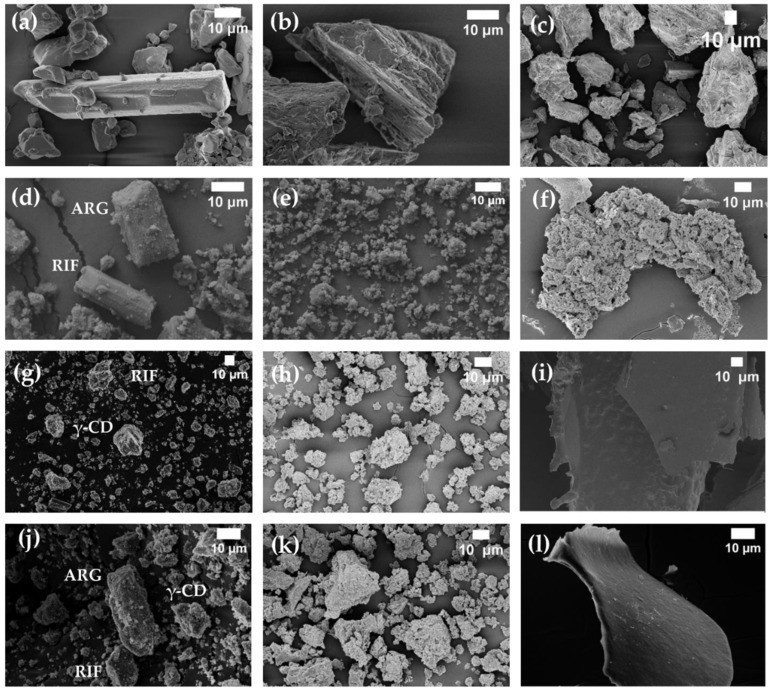
SEM images of RIF (**a**), ARG (**b**), γ-CD (**c**), RIF:ARG PM (**d**), RIF:ARG KN (**e**), RIF:ARG FD (**f**), RIF:γ-CD PM (**g**), RIF:γ-CD KN (**h**), RIF:γ-CD FD (**i**), RIF:γ-CD:ARG PM (**j**), RIF:γ-CD:ARG KN (**k**), and RIF:γ-CD:ARG FD (**l**).

FT-IR (Figure 13), PXRD (Figure 14) and SEM (Figure 15) were performed to fully characterize the complexes in the solid state. For these studies, the pure components, BC (RIF:ARG and RIF:γ-CD) and MC (RIF:γ-CD:ARG), prepared by PM, KN and FD, were analyzed and compared. As is known, RIF present two crystal forms, namely, I and II [34]. All of the techniques used in the RIF pure characterization in our investigation yielded results indicating the presence of form I. In these experiments, no differences in the behavior of BC or MC prepared by PM were found when compared to pure materials, implying that there were no interactions between the compounds in these solid systems. On the other hand, when the results for BC and MC synthesized via the KN and FD procedures were compared to the pure materials, a notable difference was detected. The characteristic bands of RIF in the infrared spectra were 3482 cm^−1^, 2980 cm^−1^, 1724 cm^−1^, 1643 cm^−1^, and 1560 cm^−1^ for -OH groups, N-CH_3_ groups, acetyl C=O, furanone C=O, and amide C=O, respectively. The FTIR spectrum of γ-CD recorded bands at 3272 cm^−1^, 2923 cm^−1^, 1637 cm^−1^, 1152 cm^−1^, and 1019 cm^−1^ due to vibrations of the O–H, C–H, H–O–H, C–O–C, and C–O groups. ARG showed characteristic bands at 3038 cm^−1^, 2944 cm^−1^, 1673 cm^−1^, and 1550 cm^−1^ for the -NH, -CH_3_, -NH_2_, and C=O groups, respectively. Carbonyl group stretching (1724 cm^−1^) was the most modified RIF band due to complex formation. In the case of the systems prepared by KN and FD, a notable reduction of the acetyl C=O band suggests the interaction between RIF, γ-CD and ARG, which was consistent with inclusion mode predicted by molecular modeling results. PXRD patterns evidenced the crystalline nature of RIF (Form I), ARG, and γ-CD. For RIF:ARG KN, RIF:γ-CD KN, and RIF:γ-CD:ARG KN, the PXRD patterns show both sharp (corresponding to Form I) and broad features (halo typical of the amorphous state), indicating that the solid produced using this method is a combination of crystalline and amorphous material. In contrast, a complete amorphization was observed for RIF:ARG FD, RIF:γ-CD FD and RIF:γ-CD:ARG FD as a broad halo in the diffraction patterns. Finally, SEM analyses were carried out to study the morphology of the particles. RIF displayed polyhedrons and column-shaped particles, γ-CD showed irregular particles of different sizes, and ARG had a large particle size and a rough surface. These results were in agreement with PXRD analyses that evidenced the crystalline nature of RIF, ARG, and γ-CD. In the PM (RIF:ARG, RIF:γ-CD and RIF:γ-CD:ARG), the characteristic morphology for the pure components was not modified. On the other hand, the RIF:ARG KN, RIF:γ-CD KN, and RIF:γ-CD:ARG KN systems recorded a notable change in the morphology and a reduction in particle size with respect to pure components, as expected given the kneading process to which they were subjected. After the FD process, RIF:ARG FD exhibited a clearly different morphology than RIF:γ-CD FD and RIF:γ-CD:ARG FD. In the case of RIF:ARG FD, agglomerated particles with some thin layers adhered to their surface were observed. By contrast, the RIF:γ-CD FD and RIF:γ-CD:ARG FD particles showed changes in the morphology, giving rise to thin layers of irregular size and shape. To conclude, the results obtained after the characterization of the solids obtained demonstrated the formation of amorphous solids in the systems prepared by the FD method. 

### 3.5. Dissolution Studies

The percentages of dissolution of RIF as a function of time for pure RIF, RIF:ARG, RIF:γ-CD, and RIF:γ-CD:ARG prepared by PM, KN, and FD are shown in Table 6. The profile of pure RIF in the dissolution medium (pH 6.8 simulated intestinal fluid) exhibited a release percentage of 14 ± 2%, 30 ± 2%, 46 ± 3%, and 63 ± 1% at 15, 30, 60, and 120 min, respectively. In relation to the system formed by RIF and ARG, the system prepared by the FD method (63 ± 3%) achieved a higher dissolution rate compared to the RIF:ARG PM (45 ± 2%) and RIF:ARG KN (51 ± 4%) systems at the beginning of the study. After 120 min, the RIF:ARG PM, RIF:ARG KN, and RIF:ARG FD systems reached RIF dissolution percentages of 94 ± 1%, 94 ± 1%, and 78 ± 1%. These results indicate that the addition of ARG increased the dissolution rate of the drug. Related to RIF:γ-CD, the solid obtained by the KN method produced a higher dissolution rate compared to the PM and FD processes, starting from 68 ± 1% to 82 ± 3% of drug dissolution. For the MC, RIF:γ-CD:ARG FD system reached the highest dissolved percentage of RIF (93 ± 3%) at 120 min. To continue with the analysis, the values of f_2_ were calculated and displayed in Table 6. From these findings, RIF:ARG, RIF:γ-CD, and RIF:γ-CD:ARG recorded f_2_ values less than 50, which indicated that the profiles for the developed systems prepared by PM, KN and FD are statistically different from the pure RIF dissolution profile. In conclusion, the better results were obtained for the RIF:ARG system prepared by PM and KN methods and for the RIF:γ-CD:ARG system prepared by FD.

### 3.6. Microbiological Studies

#### 3.6.1. In Vitro Antimicrobial Study

Assays in broth dilution showed that the MIC of RIF for MRSA 771 was 4 µg/mL. The antimicrobial activity of RIF, ARG, γ-CD, the BC, and MC determined by the diffusion method showed that ARG and γ-CD have no antimicrobial activity while RIF, the BC, and the MC have activity against MRSA 771. The inhibition halo values were 37.7 ± 0.6, 38.7 ± 0.6, 40.0 ± 0.1, and 39.7 ± 0.6 for RIF, RIF:γ-CD, RIF:ARG, and RIF:-CD:ARG, respectively. These assays demonstrate that there were significant differences (*p* < 0.05) between the pure RIF and RIF:ARG BC and the RIF:γ-CD:ARG MC inhibition zones. These results allow us to conclude that these systems exhibited significantly enhanced antibacterial properties compared to pure RIF.

#### 3.6.2. Evaluation of the Metabolic Activity of the Biofilm

The evaluation of the metabolic activity by means of the XTT assay, allowed us to demonstrate that MRSA 771 has a high capacity to produce biofilm (OD_490_ nm of 3.1 ± 0.2), which makes it an attractive strain to be studied. Furthermore, it was found that pure γ-CD and ARG do not affect biofilm metabolism.

#### 3.6.3. Biofilm Analysis by SEM

**Figure 16:**
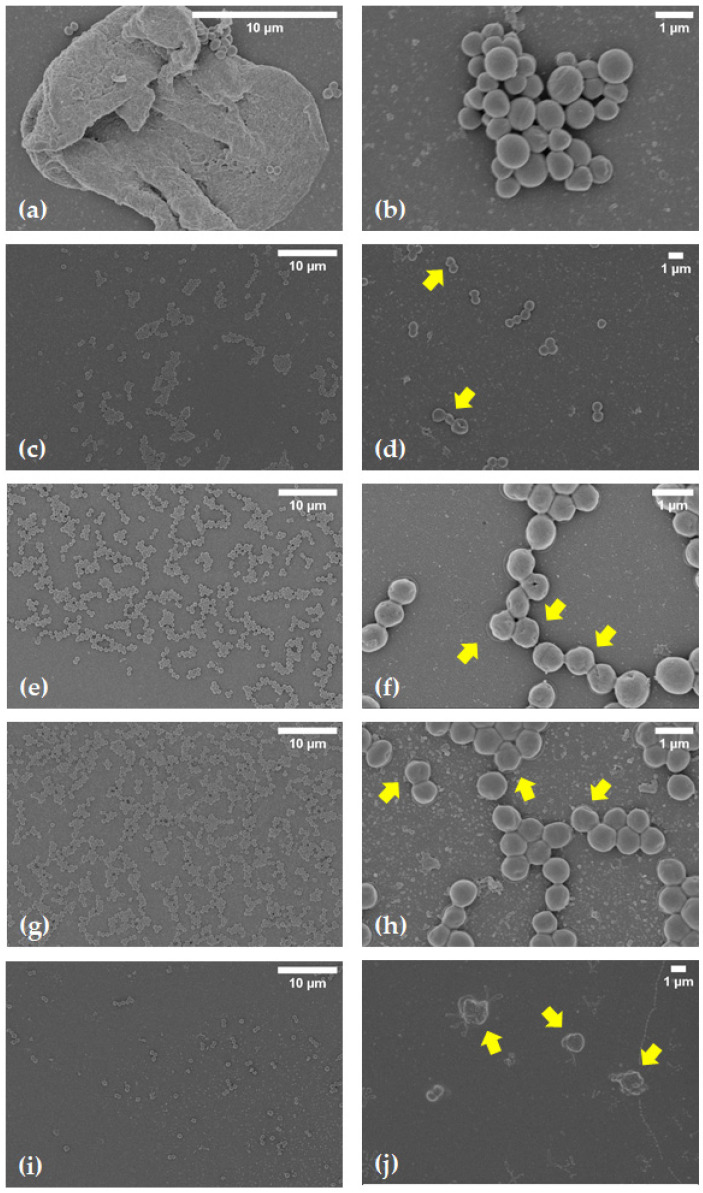
SEM images of MRSA 771 biofilm. (**a**,**b**): control without treatment, (**c**,**d**): biofilm treatment with pure RIF, (**e**,**f**): biofilm treatment with RIF:ARG, (**g**,**h**): biofilm treatment with RIF:γ-CD and (**i**,**j**): biofilm treatment with RIF:γ-CD:ARG. Yellow arrows point to damaged cells.

Given that the MIC of MRSA 771 is 4 µg/mL and knowing that biofilms are 100–1000 times more resistant to antimicrobials than their planktonic counterpart, the biofilms formed were treated with 4 mg/mL of RIF. SEM analysis (Figure 16) showed that pure RIF, RIF:γ-CD and RIF:ARG did not cause a significant decrease in cell adhesion, although cell damage could be observed (yellow arrows). On the other hand, the RIF:γ-CD:ARG MC showed good antibiofilm activity with significant damage to the bacterial cell.

### 3.7. Antileishmanial Activity

**Figure 17:**
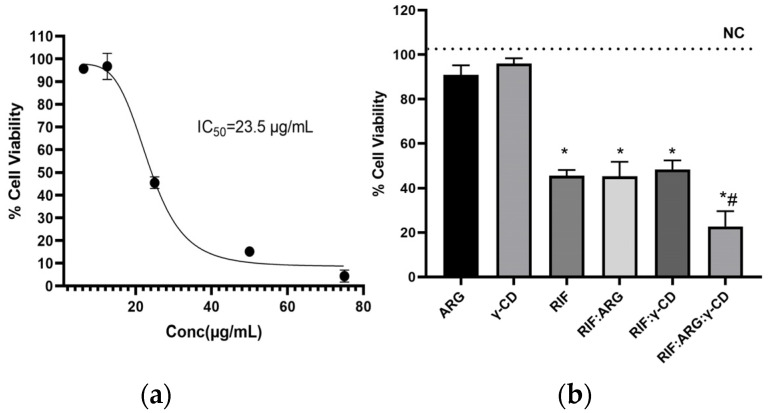
(**a**) Dose-response fitting curve of pure RIF on *L. amazonensis* promastigotes. (**b**) Analysis of the cell viability of *L. amazonensis* promastigotes after treatment with pure RIF, ARG and γ-CD and the different systems. Statistical differences are marked with symbols: (* compared to the control; # compared to pure RIF, *p* ≤ 0.05).

**Figure 18:**
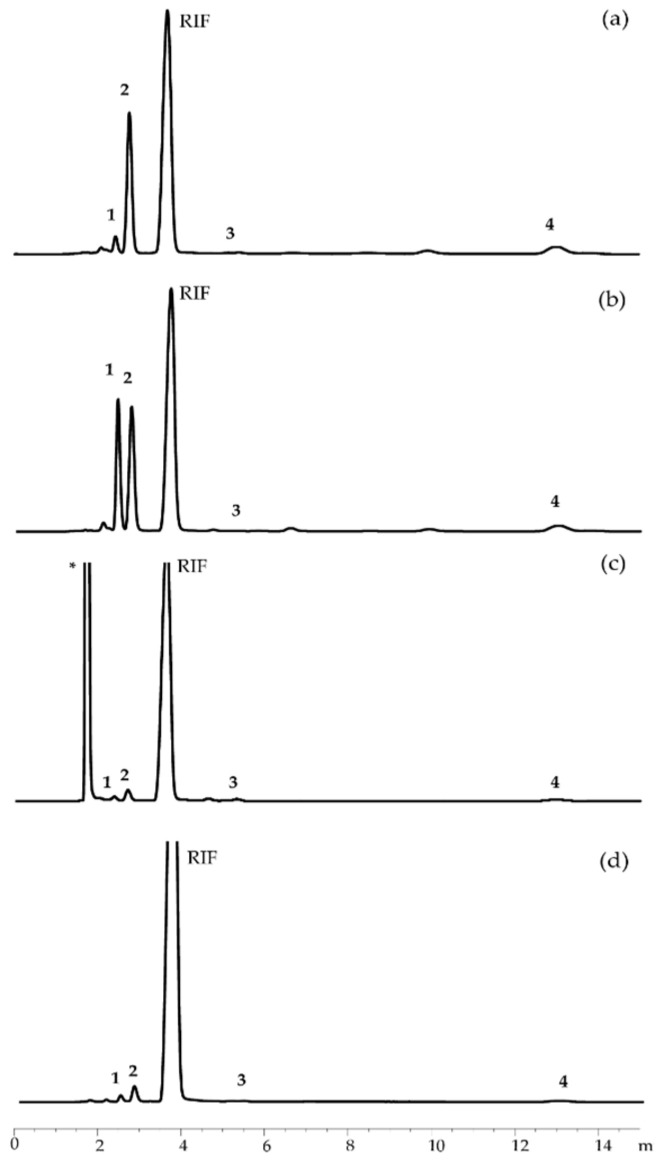
RIF chromatograms after degradation by acidic (**a**), alkaline (**b**), and oxidation (**c**) conditions, and RIF control (**d**); with the degradation products: RQ (1), SV (2), RNO (3), and 3-FR (4). * Peak corresponding to an unknown impurity in the sample.

Figure 17 shows the results of the pure RIF, the BC (RIF:ARG and RIF:γ-CD) and MC (RIF:γ-CD:ARG) comparison assay on the proliferation of *L. amazonensis* promastigotes. The IC50 of pure RIF was 23.5 µg/mL (Figure 18a). In Figure 18b it is shown that γ-CD and ARG alone had no effect on the viability of promastigote cells (*p* > 0.05 vs. NC). Treatment with pure RIF (46 ± 4%), RIF:ARG (45 ± 11%) and RIF:γ-CD (48 ± 7%) resulted in a similar reduction of % CV with statistically significant differences when compared to the NC (*p* < 0.05). It is important to note that the RIF:γ-CD:ARG MC (23 ± 12%) achieves a significant reduction in % CV when compared to pure RIF (*p* > 0.05).

### 3.8. Stability Studies

#### 3.8.1. Method Validation 

The forced degradation studies allowed us to determine the degradation products of RIF under the different stress conditions, and were used to demonstrate selectivity of the method (Figure 18). As previously reported in other research [32], the degradation products identified for RIF were: rifampicin quinone (RQ), rifamycin SV (SV), rifampicin N-oxide (RNO), and 3-formylrifamycin SV (3-FR). The data for the validation parameters listed in Table 7 demonstrated that the HPLC analytical procedure developed complied with the parameters of specificity, linearity, accuracy, and precision, and can be considered indicative of the stability.

#### 3.8.2. Stability Evaluation

Drug-cyclodextrin interactions can lead to a variety of effects on drug stability; in this sense, cyclodextrins can slow down, increase, or not affect drug degradation [35]. To evaluate the effect of complexation on drug stability, chemical stability assays on pure RIF and on the developed systems were carried out at pH 6.8. The analysis of the results exposed pseudo first-order degradation kinetics for RIF in the presence and absence of ARG and γ-CD, demonstrating that the presence of these compounds did not influence the reaction order or the degradation mechanism. The corresponding degradation rates (k_c_) and shelf-life time (t_90_) for pure RIF and BC and MC were calculated (Table 8). Drug stability was shown to be decreasing in the RIF:γ-CD system. On the other hand, the k_c_ values for RIF:ARG and RIF:γ-CD:ARG did not show significant statistical changes (*p* ≤ 0.05) with respect to pure RIF, which indicated that the formation of these systems does not negatively influence the stability of the drug. 

## 4. Conclusions

In this article, we reported the development and evaluation of the performance of RIF:γ-CD:ARG MC, which was prepared by the KN and FD methods. The formation of stable interactions between RIF, γ-CD and ARG was demonstrated by combining ^1^H NMR and molecular modeling studies. In this complex, RIF is included in the γ-CD cavity while ARG interact with the outer side surface of the macromolecule. Further, the FTIR studies showed molecular interactions between the components, the PXRD, and SEM studies, evidencing a combination of crystalline and amorphous material in the solid prepared by KN and a complete amorphization of the solid obtained by FD. The formation of RIF:γ-CD:ARG MC allowed for a greater improvement in RIF solubility and dissolution rate than the previously-reported RIF:β-CD:ARG MC [31]. Moreover, RIF:γ-CD:ARG MC was able to significantly enhance both the antibacterial and antileishmanial activity of RIF, and also reduce biofilms of *S. aureus* strains. Finally, the MC presents suitable stability. Consequently, the results show that this MC is a promising approach for developing therapeutic alternatives against infections caused by bacteria and protozoan parasites.

## Figures and Tables

**Table 1:** Free-energy analysis corresponding to the different binding modes of RIF to γ-CD.

Mode	VDW	ELE	PolSolv	NPolSolv	GAS	Total
mode-1	−25.46	35.33	43.41	−2.40	−60.79	−19.78
mode-2	−40.58	−33.90	47.88	−4.26	−74.49	30.87
mode-3	−42.09	−33.12	48.73	−4.99	−75.22	−31.47
mode-4	−15.17	−25.42	32.19	−1.32	−40.59	−9.72
mode-5	−47.91	−40.29	62.92	−5.02	−88.20	−30.30
mode-6	−32.87	−28.61	46.24	−3.78	−61.49	−19.03

**Table 2:** Free-energy analysis for the two binding modes of ARG to γ-CD.

Mode	VDW	ELE	PolSolv	NPolSolv	GAS	Total
mode-1	−6.87	−39.70	38.84	−1.15	−46.57	−8.89
mode-2	−5.96	−38.95	37.88	−1.00	−44.92	−8.03

**Table 3:** Free-energy analysis corresponding to the free binding of ARG to RIF:γ-CD BC in mode-3.3.4.

Mode	VDW	ELE	PolSolv	NPolSolv	GAS	Total
mode-3.3.4	−46.43	−25.98	44.90	−5.38	−72.41	−32.89

**Table 4:** Free-energy analysis corresponding to the interaction between ARG and RIF.

	VDW	ELE	PolSolv	NPolSolv	GAS	Total
RIF:ARG	−11.10	−38.02	42.45	−1.58	−49.13	−8.26

**Table 5:** Chemical shift displacements for the RIF:ARG, RIF:γ-CD, and RIF:γ-CD:ARG systems.

^1^H-NMR Signals	Δ𝛅 ppm		
RIF Protons	RIF:ARG	RIF:γ-CD	RIF:γ-CD:ARG
H33	−0.0400	−0.0470	−0.0535
H31	−0.0265	−0.0255	−0.0645
H32	−0.0290	−0.0310	−0.0670
H26	−0.0240	−0.0405	−0.0670
H24	−0.0360	−0.0455	−0.0645
H13	−0.0300	−0.0640	−0.1330
H22	0.0175	−0.0060	0.0845
H14	−0.0290	−0.0190	−0.0430
H30	−0.0130	−0.0440	−0.0440
H36	−0.0310	−0.0360	−0.0630
H20	−0.0255	−0.0245	−0.0695
H4’	0.0360	0.1280	−0.3325
H37	−0.0320	−0.0335	−0.0550
H3’–H5’	−0.0015	−0.0220	−0.0685
H2’–H6’	−0.0750	−0.0800	−0.0315
H27	−0.0295	−0.0335	−0.0725
H21	−0.0310	0.0060	−0.0260
H28	−0.0175	0.0055	−0.0795
H19	0.0000	−0.0065	−0.0845
H29	0.0905	−0.0265	−0.0720
H17	0.0205	−0.0125	−0.0715
H18	0.0035	0.0650	−0.0830
H1′	−0.0180	0.0170	−0.4670
γ-CD protons			
H4	-	0.0020	−0.0235
H2	-	−0.0015	−0.0380
H5–H6	-	−0.0140	−0.0760
H3	-	−0.0040	−0.0360
H1	-	−0.0060	−0.0470
ARG protons			
H4–H3	0.0345	-	0.0395
H5	0.0015	-	−0.0410
H2	−0.0250	-	−0.0785

**Table 6:** Percentages of dissolved RIF (%) at 15, 30, 60, and 120 min of the RIF:ARG, RIF:γ-CD, and RIF:γ-CD:ARG systems prepared by PM, K, and FD.

	RIF Dissolved (%) at Each Sampling Time				f_2_ Values
	15 min	30 min	60 min	120 min	
Pure RIF	14 ± 2%	30 ± 2%	46 ± 3%	63 ± 1%	
RIF:ARG	15 min	30 min	60 min	120 min	f_2_
PM	45 ± 2%	71 ± 3%	91 ± 2%	94.3 ± 0.4%	23
KN	51 ± 4%	70 ± 2%	84 ± 3%	94 ± 1%	24
FD	63 ± 3%	73 ± 1%	79 ± 1%	78 ± 1%	24
RIF:γ-CD	15 min	30 min	60 min	120 min	f_2_
PM	31 ± 1%	52 ± 3%	71 ± 2%	80 ± 1%	35
KN	68 ± 1%	80 ± 1%	80 ± 2%	82 ± 3%	22
FD	35 ± 2%	50 ± 3%	61 ± 2%	74 ± 2%	41
RIF:γ-CD:ARG	15 min	30 min	60 min	120 min	f_2_
PM	43 ± 1%	58 ± 3%	68 ± 2%	71 ± 3%	34
KN	67 ± 2%	67 ± 5%	68 ± 2%	68 ± 1%	26
FD	42 ± 1%	63 ± 2%	83 ± 7%	93 ± 3%	26

**Table 7:** Summary of validation results for HPLC method for determination of RIF.

Parameter	Acceptance Criteria	Calculated Values
Linearity	r^2^ > 0.998	Range = 50–150 µg/mL Area = 29.627 × C (µg/mL) − 42.365 r^2^ = 0.9996
Accuracy	Recovery 98–102%	n = 9 RIF (µg/mL) = 50, 100, 150 Average recovery (%) = 100, 100, 99
Repeatability	CV < 2%	n = 9 RIF (µg/mL) = 50, 100, 150 CV (%) = 0.01, 0.38, 0.88
Intermediate precision	CV < 3%	n = 9 RIF (µg/mL) = 50, 100, 150 CV (%) = 0.01, 1.19, 0.33
DL	DL << 0.05%	DL = 0.001% (0.002 µg/mL)
QL	QL ≤ 0.05%	QL = 0.004% (0.006 µg/mL)

**Table 8:** Kinetic parameters obtained for RIF, BC and MC in buffer solution (pH 6.8).

Solutions	k_c_ × 10^−3^ (h^−1^)	t_90_ (h)
RIF	30 ± 1	3.5 ± 0.1
RIF:ARG	32.0 ± 0.4	3.28 ± 0.04
RIF:γ-CD	37.1 ± 0.4	2.88 ± 0.03
RIF:γ-CD:ARG	31.9 ± 0.1	3.29 ± 0.01

## Data Availability

The data presented in this study are available in the article and Supplementary File.

## Supplementary Materials

The following supporting information can be downloaded at: https://www.mdpi.com/article/10.3390/pharmaceutics15010198/s1, Figure S1: Phase solubility diagrams of the binary (RIF:γ-CD, squares) and multicomponent (RIF:γ-CD:ARG, circles) complexes; Figure S2: Distance between the centers of mass of the guest (RIF) and host (γ-CD) modecules within the MD trajectory corresponding to binding mode-3; Figure S3: Distance between the centers of mass of the guest (RIF) and host (γ-CD) modecules within the MD trajectory corresponding to binding mode-5; Figure S4: Distance between the centers of mass of the guest (ARG) and host (γ-CD) modecules within the MD trajectory corresponding to binding mode-1; Figure S5: Distance between the centers of mass of the guest (ARG) and host (γ-CD) modecules within the MD trajectory corresponding to binding mode-2; Figure S6: Structural analysis on the formation of the ternay complex mode-3.1.1; Figure S7: Structural analysis on the formation of the ternay complex mode-3.1.2; Figure S8: Structural analysis on the formation of the ternay complex mode-3.1.3; Figure S9: Structural analysis on the formation of the ternay complex mode-3.2.1; Figure S10: Structural analysis on the formation of the ternay complex mode-3.2.2; Figure S11: Structural analysis on the formation of the ternay complex mode-3.3.1; Figure S12: Structural analysis on the formation of the ternay complex mode-3.3.2; Figure S13: Structural analysis on the formation of the ternay complex mode-3.3.3; Figure S14: Structural analysis on the formation of the ternay complex mode-3.3.4; Figure S15: Structural analysis on the formation of the ternay complex mode-3.3.5; Figure S16: Structural analysis on the formation of the ternay complex mode-3.3.6; Figure S17: Structural analysis on the formation of the ternay complex mode-5.1.1; Figure S18: Structural analysis on the formation of the ternay complex mode-5.1.2; Figure S19: Structural analysis on the formation of the ternay complex mode-5.2.1; Figure S20: Structural analysis on the formation of the ternay complex mode-5.2.2; Figure S21: Structural analysis on the formation of the ternay complex mode-5.2.3; Figure S22: Structural analysis on the formation of the ternay complex mode-5.3.1; Figure S23: Structural analysis on the formation of the ternay complex mode-5.3.2; Figure S24: Structural analysis on the formation of the ternay complex mode-5.3.3; Figure S25: Temperature profile for the γ-CD systems set at 290 K; Figure S26: Temperature profile for the γ-CD systems set at 293 K; Figure S27: Temperature profile for the γ-CD systems set at 295 K; Figure S28: Temperature profile for the γ-CD systems set at 298 K; Table S1: Chemical shift for the RIF, γ-CD and ARG in free and complex forms.

## References

[1] Rohr J.R., Barrett C.B., Civitello D.J., Craft M.E., Delius B., DeLeo G.A., Hudson P.J., Jouanard N., Nguyen K.H., Ostfeld R.S. (2019). Emerging human infectious diseases and the links to global food production. Nat. Sustain..

[2] Sartini S., Permana A.D., Mitra S., Tareq A.M., Salim E., Ahmad I., Harapan H., Emran T.B., Nainu F. (2021). Current State and Promising Opportunities on Pharmaceutical Approaches in the Treatment of Polymicrobial Diseases. Pathogens.

[3] Aiassa V., Zoppi A., Albesa I., Longhi M.R. (2015). Inclusion complexes of chloramphenicol with β-cyclodextrin and aminoacids as a way to increase drug solubility and modulate ROS production. Carbohydr. Polym..

[4] Aiassa V., Zoppi A., Becerra M.C., Albesa I., Longhi M.R. (2016). Enhanced inhibition of bacterial biofilm formation and reduced leukocyte toxicity by chloramphenicol: β-cyclodextrin: N-acetylcysteine complex. Carbohydr. Polym..

[5] Cerutti J.P., Aiassa V., Fernandez M.A., Longhi M.R., Quevedo M.A., Zoppi A. (2021). Structural, physicochemical and biological characterization of chloramphenicol multicomponent complexes. J. Mol. Liq..

[6] Zoppi A., Buhlman N., Cerutti J.P., Longhi M.R., Aiassa V. (2019). Influence of proline and β-Cyclodextrin in ketoconazole physicochemical and microbiological performance. J. Mol. Struct..

[7] Zoppi A., Bartolilla A., Longhi M.R., Aiassa V. (2020). Simultaneous improvement of ketoconazole solubility, antifungal and antibiofilm activity by multicomponent complexation. Ther. Deliv..

[8] Morin-Crini N., Fourmentin S., Fenyvesi É., Lichtfouse E., Torri G., Fourmentin M., Crini G. (2021). 130 years of cyclodextrin discovery for health, food, agriculture, and the industry: A review. Environ. Chem. Lett..

[9] Jansook P., Ogawa N., Loftsson T. (2018). Cyclodextrins: Structure, physicochemical properties and pharmaceutical applications. Int. J. Pharm..

[10] Aiassa V., Garnero C., Longhi M.R., Zoppi A. (2021). Cyclodextrin Multicomponent Complexes: Pharmaceutical Applications. Pharmaceutics.

[11] Neuber H. (2008). Leishmaniasis. J. Dtsch. Dermatol. Ges..

[12] Von Stebut E. (2015). Leishmaniasis. JDDG J. Dtsch. Dermatol. Ges..

[13] Jolivet-Gougeon A., Bonnaure-Mallet M. (2014). Biofilms as a mechanism of bacterial resistance. Drug Discov. Today Technol..

[14] Chadha R., Saini A., Gupta S., Arora P., Thakur D., Jain D.V.S. (2010). Encapsulation of rifampicin by natural and modified β-cyclodextrins: Characterization and thermodynamic parameters. J. Incl. Phenom. Macrocycl. Chem..

[15] Anjani Q.K., Domínguez-Robles J., Utomo E., Font M., Martínez-Ohárriz M.C., Permana A.D., Cárcamo-Martínez Á., Larrañeta E., Donnelly R.F. (2021). Inclusion Complexes of Rifampicin with Native and Derivatized Cyclodextrins: In Silico Modeling, Formulation, and Characterization. Pharmaceuticals.

[16] Dan Córdoba A.V., Aiassa V., Longhi M.R., Quevedo M.A., Zoppi A. (2020). Improved Activity of Rifampicin Against Biofilms of Staphylococcus aureus by Multicomponent Complexation. AAPS PharmSciTech.

[17] Higuchi T., Connors K.A. (1965). Phase-solubility techniques. Adv. Anal. Chem. Instr..

[18] https://www.ccdc.cam.ac.uk/.

[19] Kirschner K.N., Yongye A.B., Tschampel S.M., González-Outeiriño J., Daniels C.R., Foley B.L., Woods R.J. (2008). Glycam06: A generalizable biomolecular force field. Carbohydrates. J. Comput. Chem..

[20] https://chemaxon.com/marvin.

[21] He X., Man V.H., Yang W., Lee T.S., Wang J. (2020). A fast and high-quality charge model for the next generation general amber force field. J. Chem. Phys..

[22] Santos-Martins D., Solis-Vasquez L., Tillack A.F., Sanner M.F., Koch A., Forli S. (2021). Accelerating autodock4 with gpus and gradient-based local search. J. Chem. Theory Comput..

[23] Morris G.M., Huey R., Olson A.J. (2008). Using autodock for ligand-receptor docking. Curr. Protoc. Bioinform..

[24] Miller B.R., McGee T.D., Swails J.M., Homeyer N., Gohlke H., Roitberg A.E. (2012). Mmpbsa. py: An efficient program for end-state free energy calculations. J. Chem. Theory Comput..

[25] Case D., Aktulga H., Belfon K., Ben-Shalom I., Berryman J., Brozell S., Cerutti D., Cheatham T., Cisneros G., Cruzeiro V. (2022). Amber22.

[26] Mark P., Nilsson L. (2001). Structure and dynamics of the tip3p, spc, and spc/e water models at 298 K. J. Phys. Chem. A.

[27] Gowers R.J., Linke M., Barnoud J., Reddy T.J., Melo M.N., Seyler S.L., Domanski J., Dotson D.L., Buchoux S., Kenney I.M. Mdanalysis: A python package for the rapid analysis of molecular dynamics simulations. Proceedings of the 15th Python in Science Conference.

[28] Humphrey W., Dalke A., Schulten K. (1996). Vmd: Visual molecular dynamics. J. Mol. Graph..

[29] https://ccad.unc.edu.ar/.

[30] Clinical and Laboratory Standards Institute (CLSI) (2012). Methods for Dilution Antimicrobial Susceptibility Tests for Bacteria that Grow Aerobically. Approved Standard—7th Edition.

[31] Cortez Marcolino L.M., Correia Pereira A.H., Guerra Pinto J., Mamone L.A., Ferreira Strixino J. (2021). Cellular and metabolic changes after photodynamic therapy in leishmania promastigotes. Photodiagnosis Photodyn. Ther..

[32] Liu J., Sun J., Zhang W., Gao K., He Z. (2008). HPLC determination of rifampicin and related compounds in pharmaceuticals using monolithic column. J. Pharm. Biomed. Anal..

[33] ICH Harmonised Tripartite Guideline (2005). Validation of Analytical Procedures: Text and Methodology. Q2 (R1).

[34] Jing D., Gu Y., Xia H. (2018). Solid-State and Solution-Mediated Polymorphic Transformation of Rifampicin. Chem. Eng. Technol..

[35] Popielec A., Loftsson T. (2017). Effects of cyclodextrins on the chemical stability of drugs. Int. J. Pharm..

