# Supplementary material for: Development and Characterization of Pharmaceutical Systems Containing Rifampicin
Source: Pharmaceutics. 2023 Jan 5;15(1):198. doi: 10.3390/pharmaceutics15010198 (PMC9864009; doi:10.3390/pharmaceutics15010198)
Supplement: Supplementary file 1 [file pharmaceutics-15-00198-s001.zip › pharmaceutics-2074915-supplementary.pdf]

Supplementary Material:

"Development and characterization of pharmaceutical systems containing rifampicin"

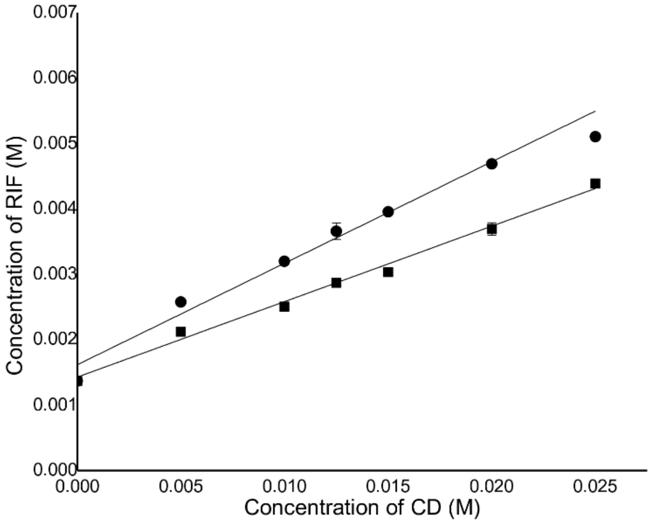

Figure S1: Phase solubility diagrams of the binary (RIF:γ-CD, squares) and multicomponent (RIF:γ-CD:ARG, circles) complexes.

1. Supplementary Material

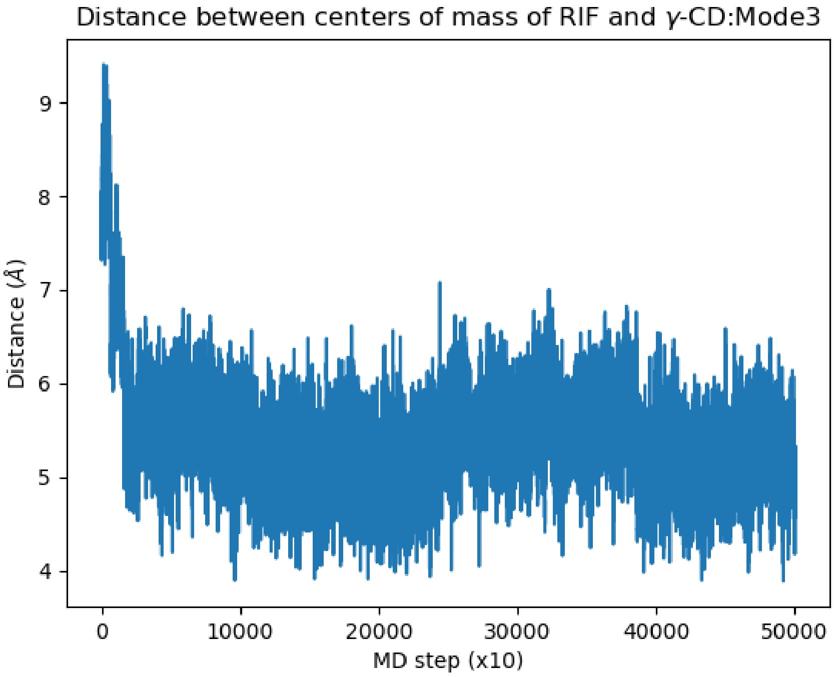

Figure S2: Distance between the centers of mass of the guest (RIF) and host (γ-CD) modecules within the MD trajectory corresponding to binding mode-3.

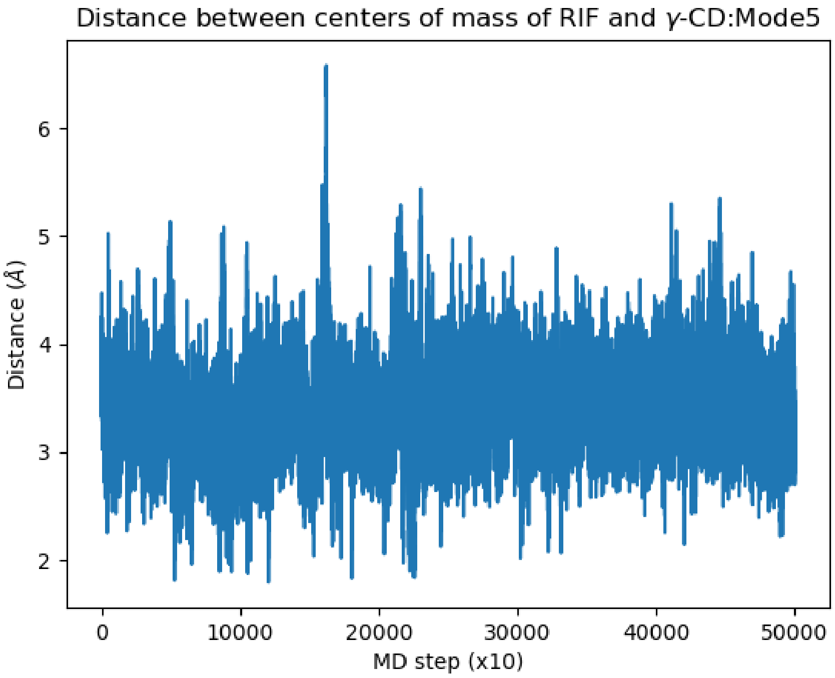

Figure S3: Distance between the centers of mass of the guest (RIF) and host (γ-CD) modecules within the MD trajectory corresponding to binding mode-5.

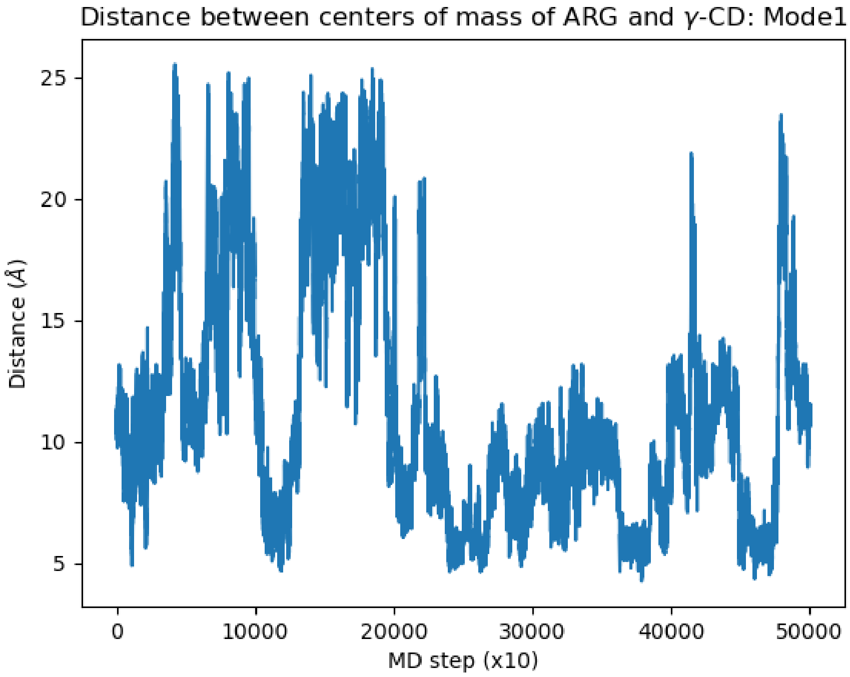

Figure S4: Distance between the centers of mass of the guest (ARG) and host (γ-CD) molecules within the MD trajectory corresponding to binding mode-1.

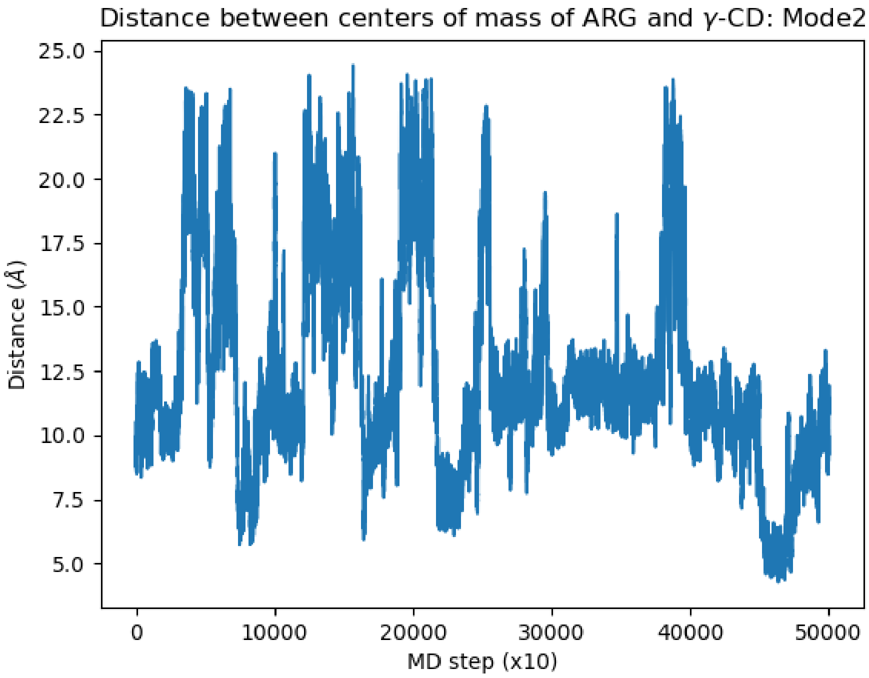

Figure S5: Distance between the centers of mass of the guest (ARG) and host (γ-CD) modecules within the MD trajectory corresponding to binding mode-2.

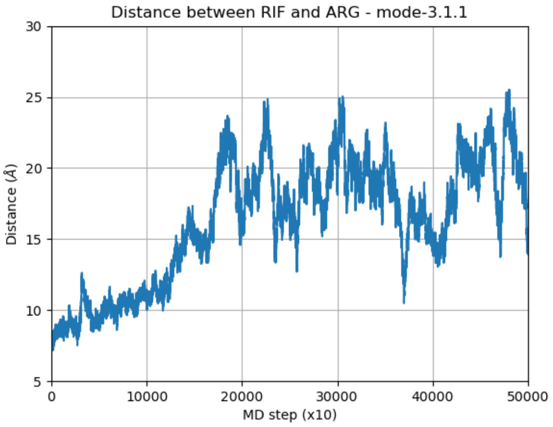

(a) *Distance between RIF and ARG in the ternary complex mode-3.1.1*

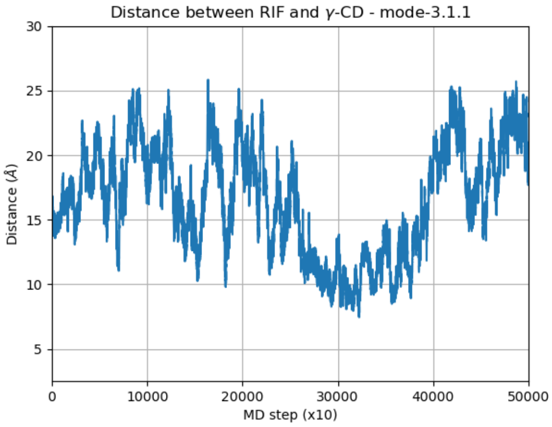

(b) *Distance between RIF and γ-CD in the ternary complex mode-3.1.1*

Figure S6: Structural analysis on the formation of the ternay complex mode-3.1.1

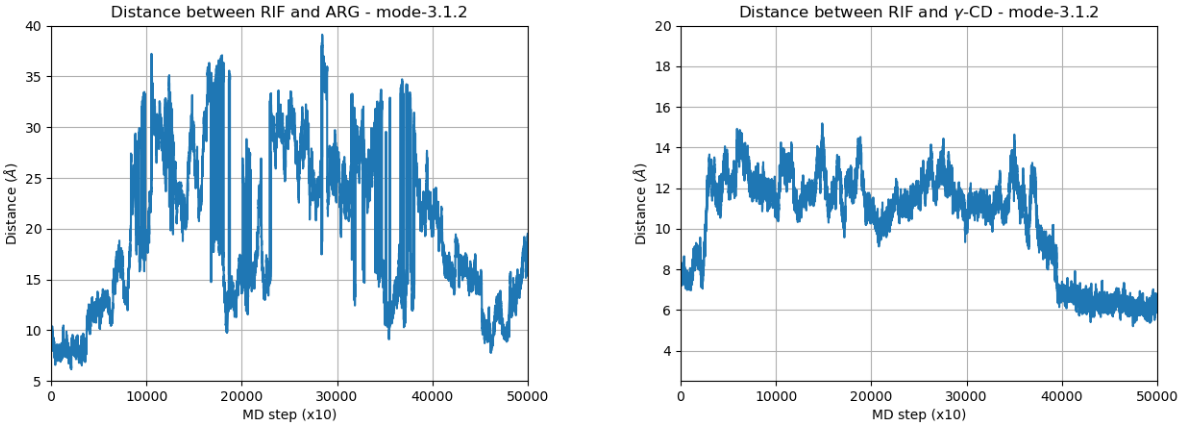

(a) *Distance between RIF and ARG in the ternary complex mode-3.1.2*

(b) *Distance between RIF and γ-CD in the ternary complex mode-3.1.2*

Figure S7: Structural analysis on the formation of the ternay complex mode-3.1.2

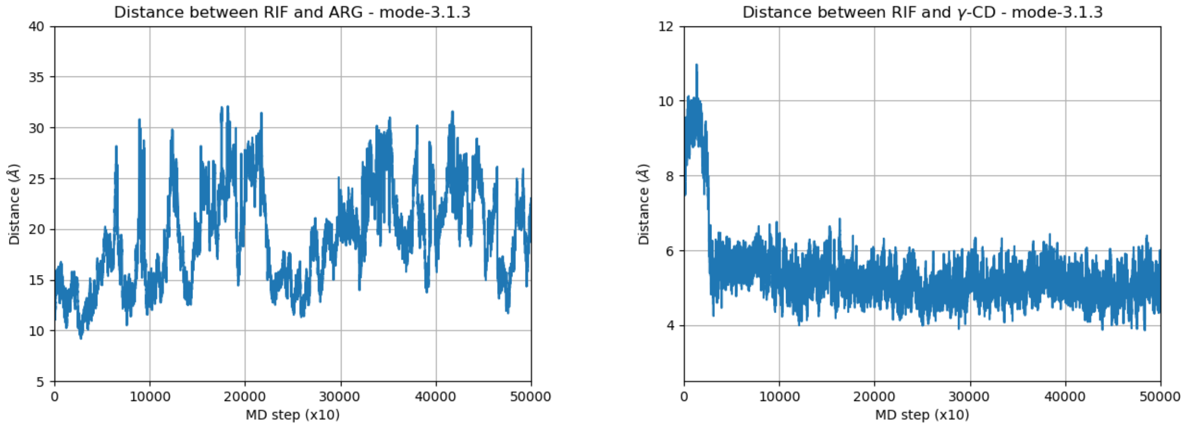

(a) *Distance between RIF and ARG in the ternary complex mode-3.1.3*

(b) *Distance between RIF and γ-CD in the ternary complex mode-3.1.3*

Figure S8: Structural analysis on the formation of the ternay complex mode-3.1.3

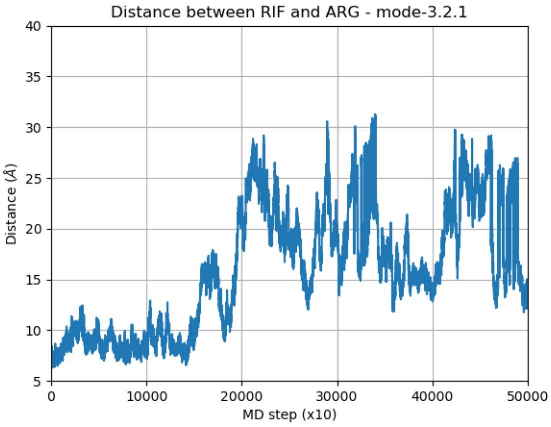

(a) *Distance between RIF and ARG in the ternary complex mode-3.2.1*

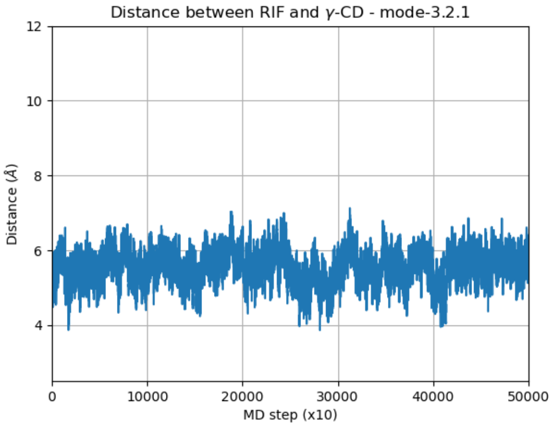

(b) *Distance between RIF and γ-CD in the ternary complex mode-3.2.1*

Figure S9: Structural analysis on the formation of the ternay complex mode-3.2.1

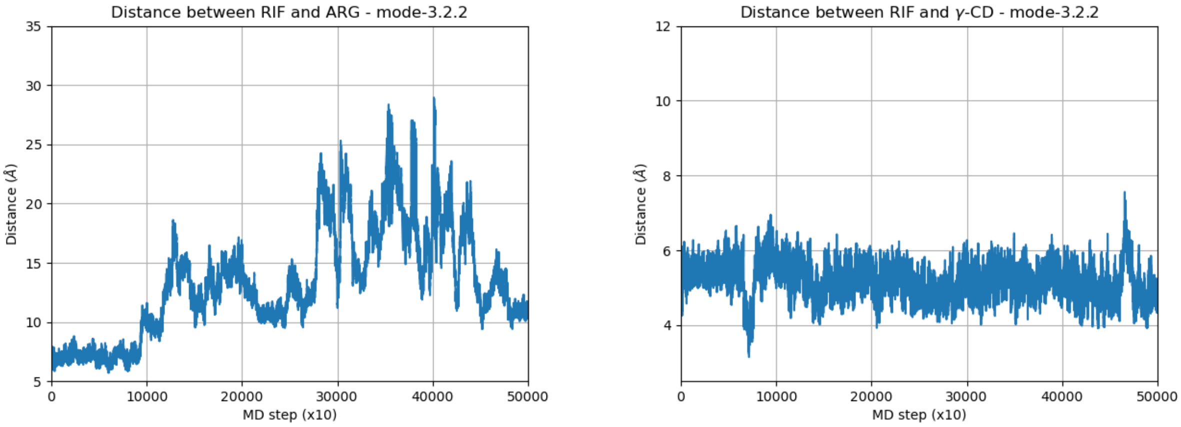

(a) *Distance between RIF and ARG in the ternary complex mode-3.2.2*

(b) *Distance between RIF and γ-CD in the ternary complex mode-3.2.2*

Figure S10: Structural analysis on the formation of the ternay complex mode-3.2.2

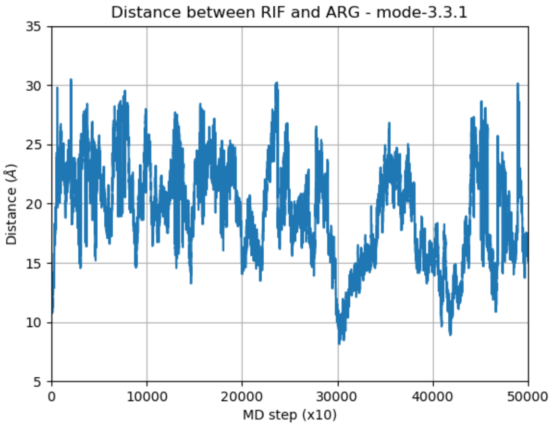

(a) *Distance between RIF and ARG in the ternary complex mode-3.3.1*

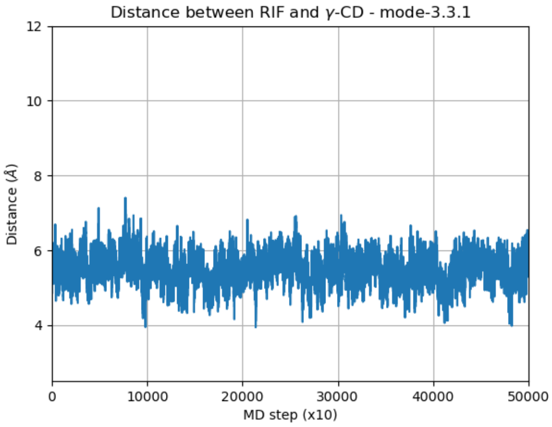

(b) *Distance between RIF and γ-CD in the ternary complex mode-3.3.1*

Figure S11: Structural analysis on the formation of the ternay complex mode-3.3.1

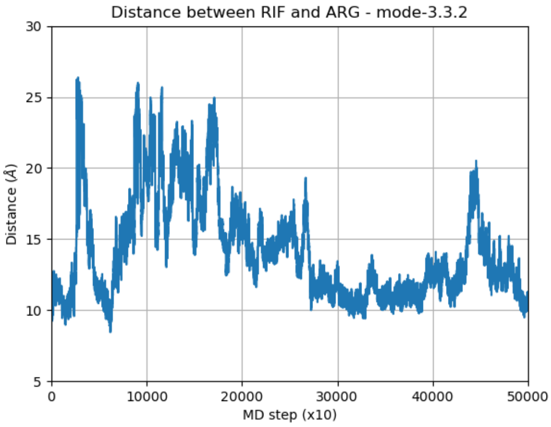

(a) *Distance between RIF and ARG in the ternary complex mode-3.3.2*

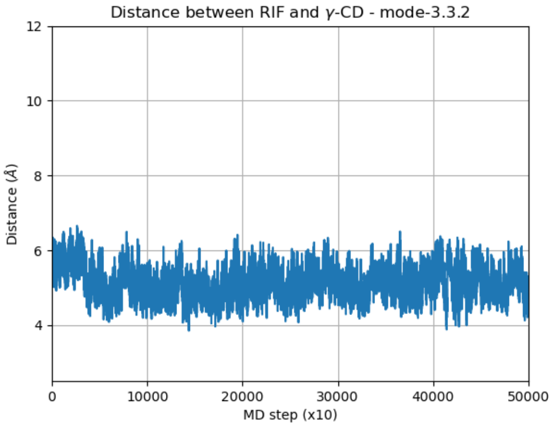

(b) *Distance between RIF and γ-CD in the ternary complex mode-3.3.2*

Figure S12: Structural analysis on the formation of the ternay complex mode-3.3.2

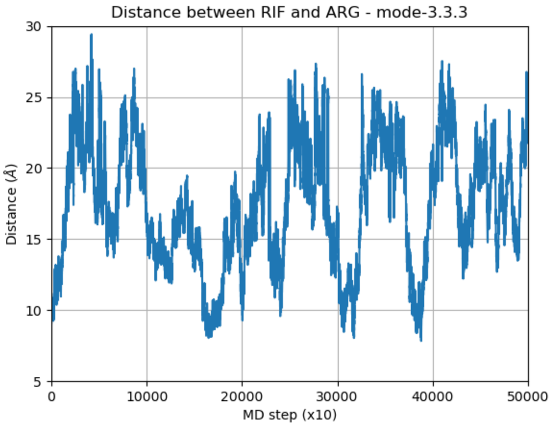

(a) *Distance between RIF and ARG in the ternary complex mode-3.3.3*

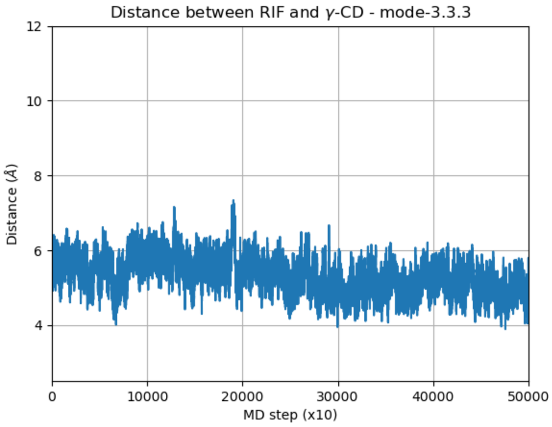

(b) *Distance between RIF and γ-CD in the ternary complex mode-3.3.3*

Figure S13: Structural analysis on the formation of the ternay complex mode-3.3.3

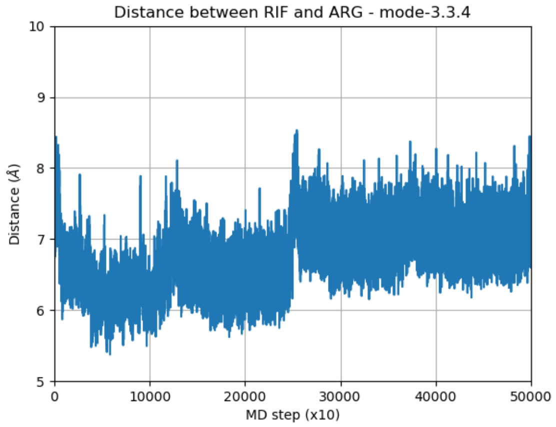

(a) *Distance between RIF and ARG in the ternary complex mode-3.3.4*

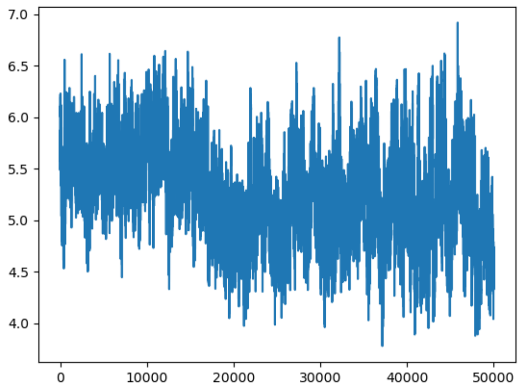

(b) *Distance between RIF and γ-CD in the ternary complex mode-3.3.4*

Figure S14: Structural analysis on the formation of the ternay complex mode-3.3.4

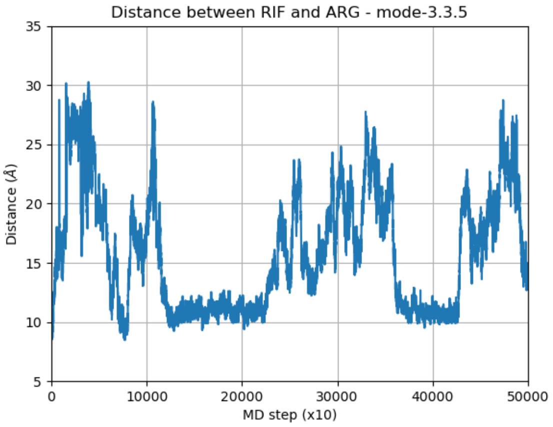

(a) *Distance between RIF and ARG in the ternary complex mode-3.3.5*

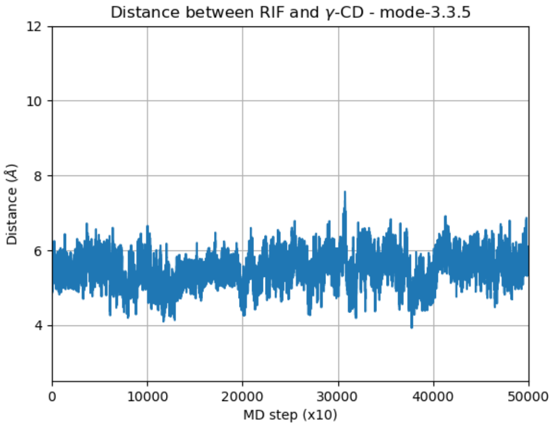

(b) *Distance between RIF and γ-CD in the ternary complex mode-3.3.5*

Figure S15: Structural analysis on the formation of the ternay complex mode-3.3.5

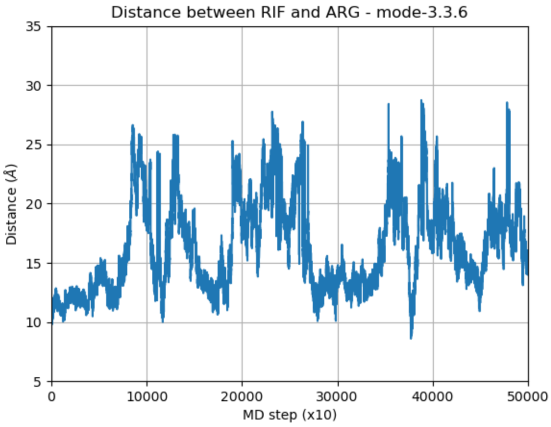

(a) *Distance between RIF and ARG in the ternary complex mode-3.3.6*

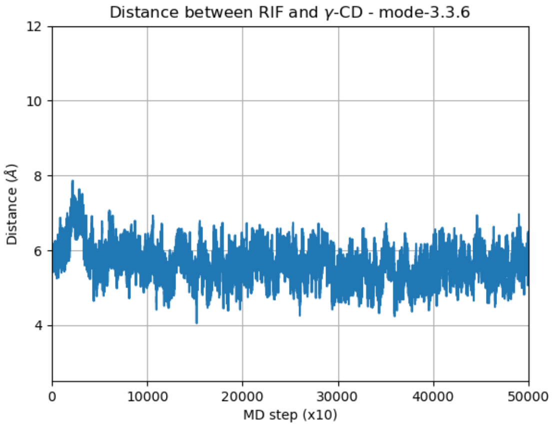

(b) *Distance between RIF and γ-CD in the ternary complex mode-3.3.6*

Figure S16: Structural analysis on the formation of the ternay complex mode-3.3.6

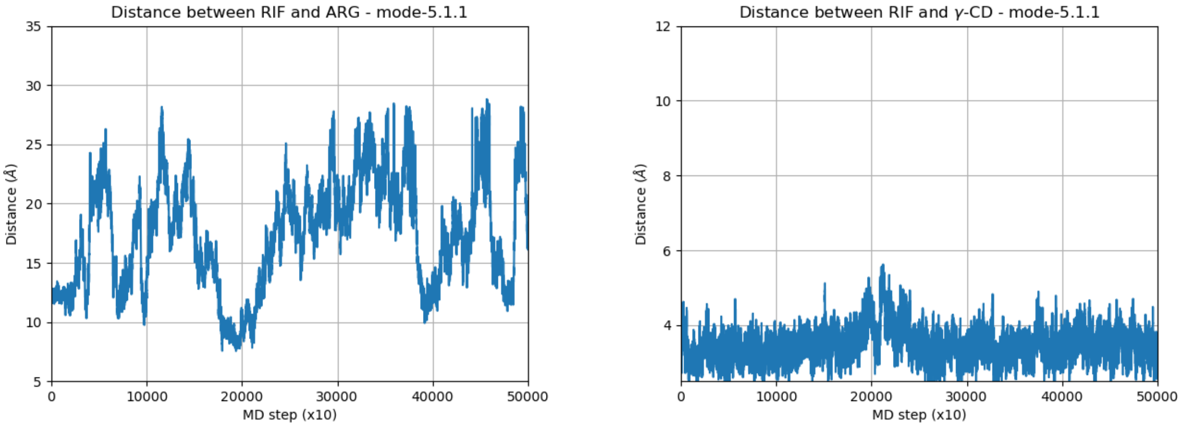

(a) *Distance between RIF and ARG in the ternary complex mode-5.1.1*

(b) *Distance between RIF and γ-CD in the ternary complex mode-5.1.1*

Figure S17: Structural analysis on the formation of the ternay complex mode-5.1.1

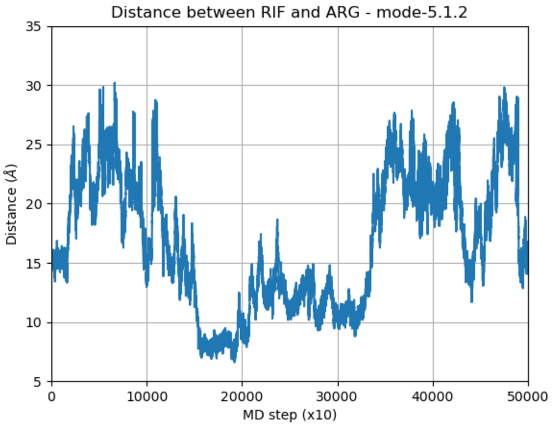

(a) *Distance between RIF and ARG in the ternary complex mode-5.1.2*

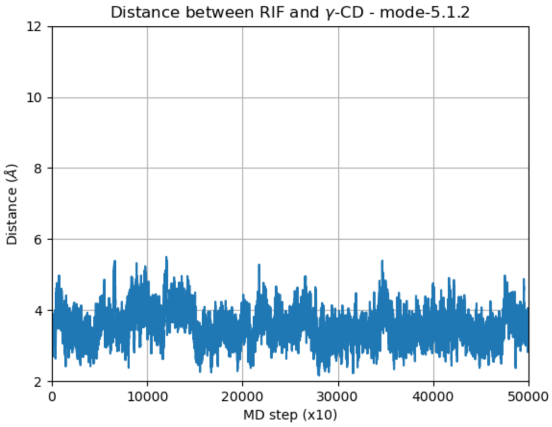

(b) *Distance between RIF and γ-CD in the ternary complex mode-5.1.2*

Figure S18: Structural analysis on the formation of the ternay complex mode-5.1.2

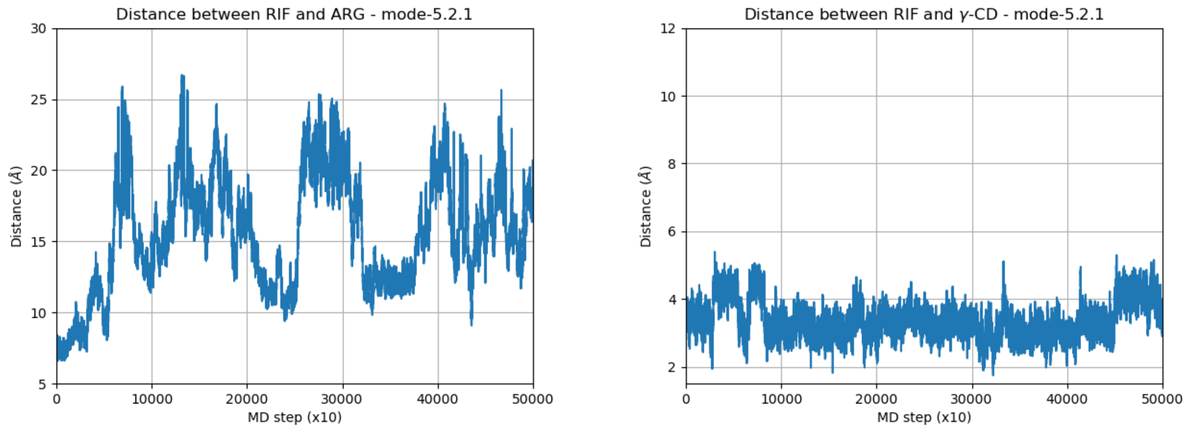

(a) *Distance between RIF and ARG in the ternary complex mode-5.2.1*

(b) *Distance between RIF and γ-CD in the ternary complex mode-5.2.1*

Figure S19: Structural analysis on the formation of the ternay complex mode-5.2.1

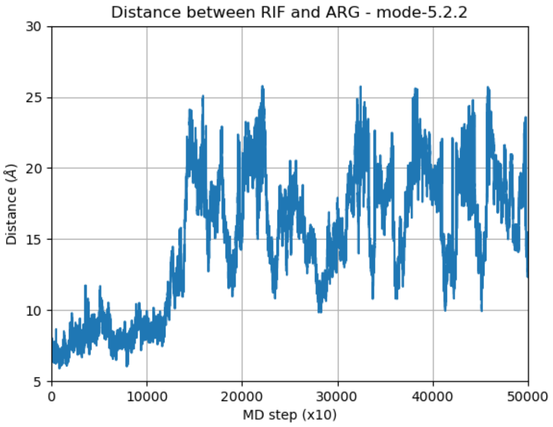

(a) *Distance between RIF and ARG in the ternary complex mode-5.2.2*

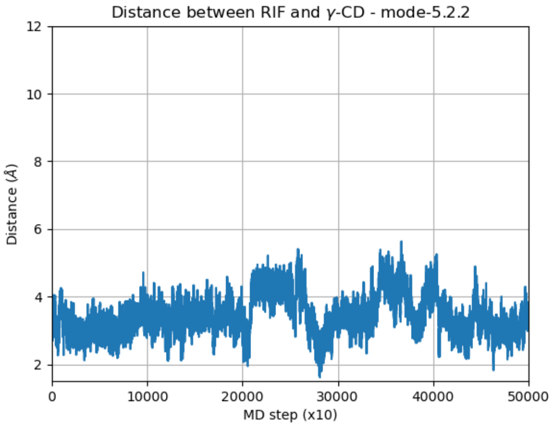

(b) *Distance between RIF and γ-CD in the ternary complex mode-5.2.2*

Figure S20: Structural analysis on the formation of the ternay complex mode-5.2.2

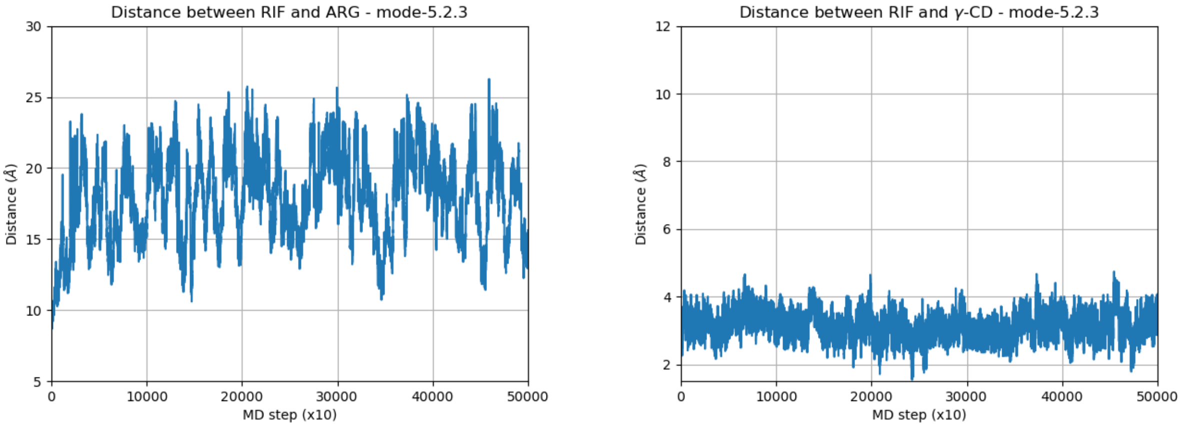

(a) *Distance between RIF and ARG in the ternary complex mode-5.2.3*

(b) *Distance between RIF and γ-CD in the ternary complex mode-5.2.3*

Figure S21: Structural analysis on the formation of the ternay complex mode-5.2.3

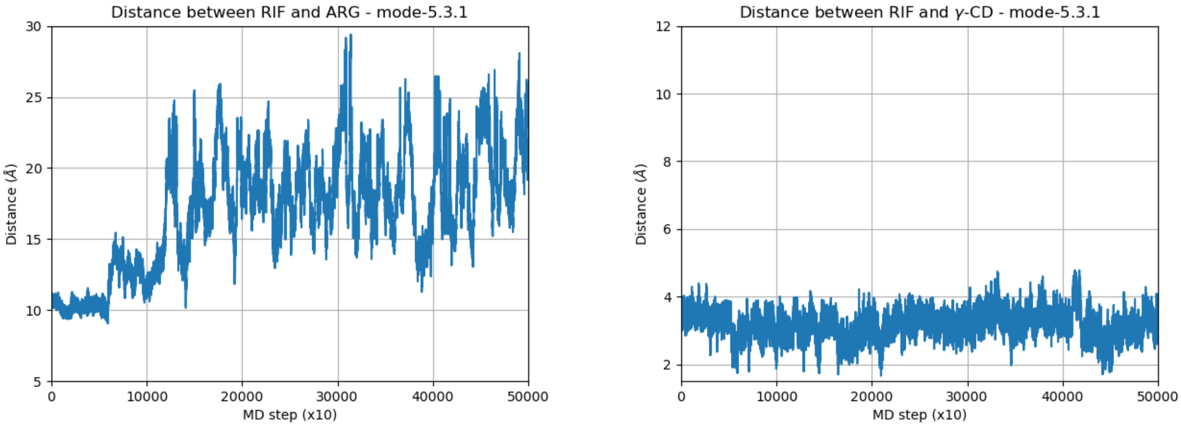

(a) *Distance between RIF and ARG in the ternary complex mode-5.3.1*

(b) *Distance between RIF and γ-CD in the ternary complex mode-5.3.1*

Figure S22: Structural analysis on the formation of the ternay complex mode-5.3.1

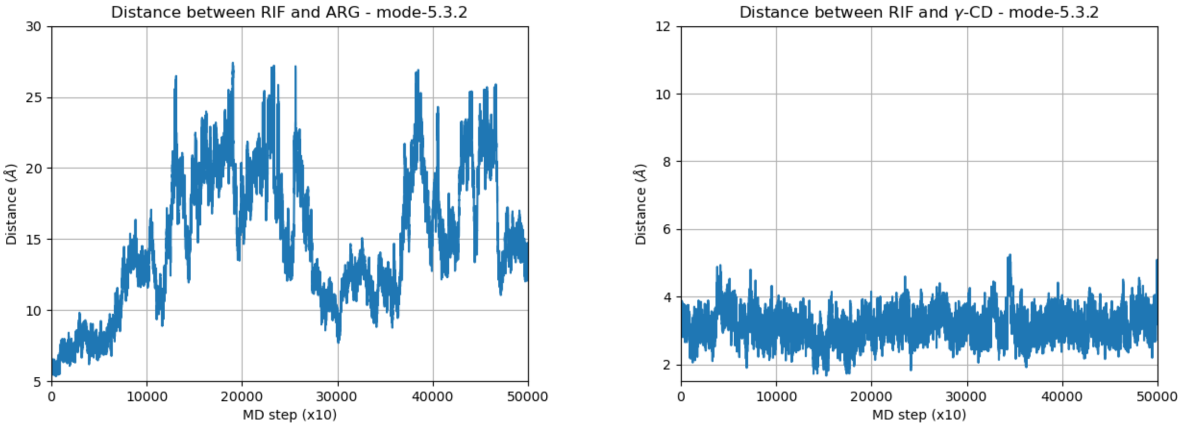

(a) *Distance between RIF and ARG in the ternary complex mode-5.3.2*

(b) *Distance between RIF and γ-CD in the ternary complex mode-5.3.2*

Figure S23: Structural analysis on the formation of the ternay complex mode-5.3.2

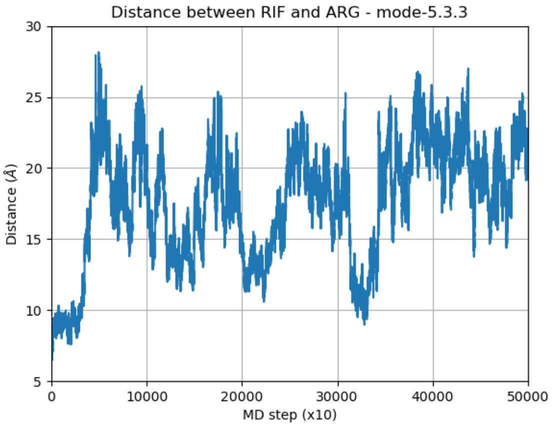

(a) *Distance between RIF and ARG in the ternary complex mode-5.3.3*

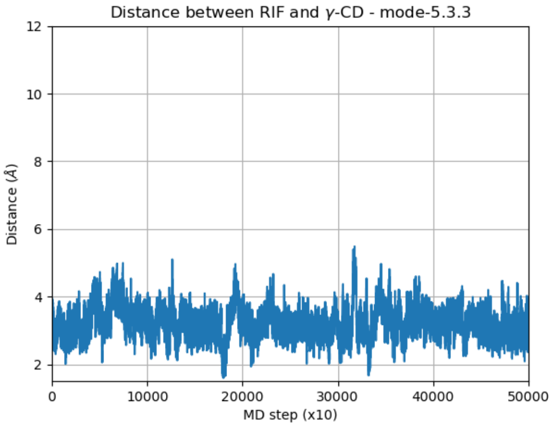

(b) *Distance between RIF and γ-CD in the ternary complex mode-5.3.3*

Figure S24: Structural analysis on the formation of the ternay complex mode-5.3.3

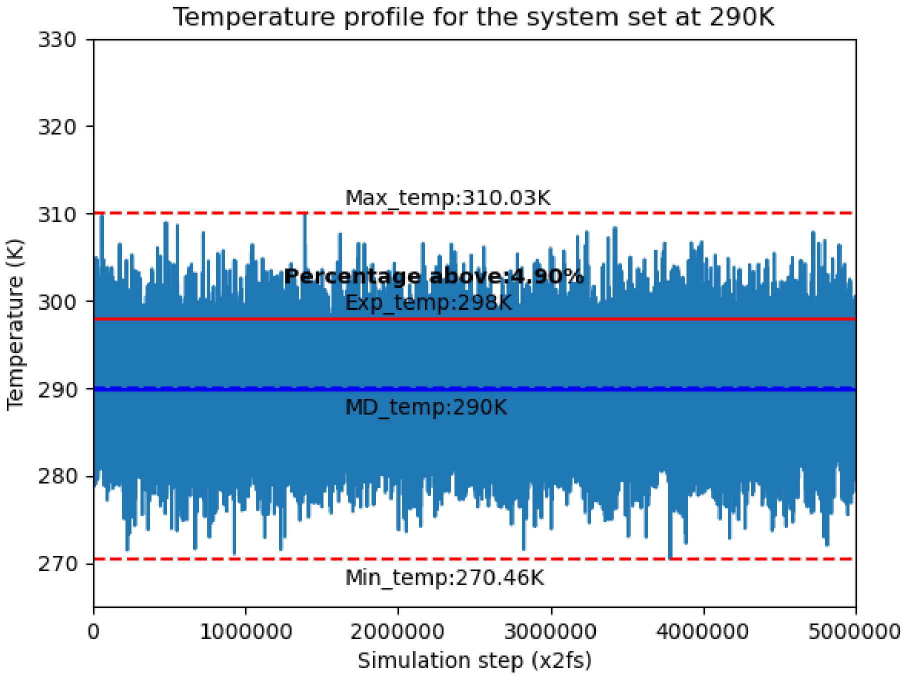

Figure S25: Temperature profile for the γ-CD systems set at 290K.

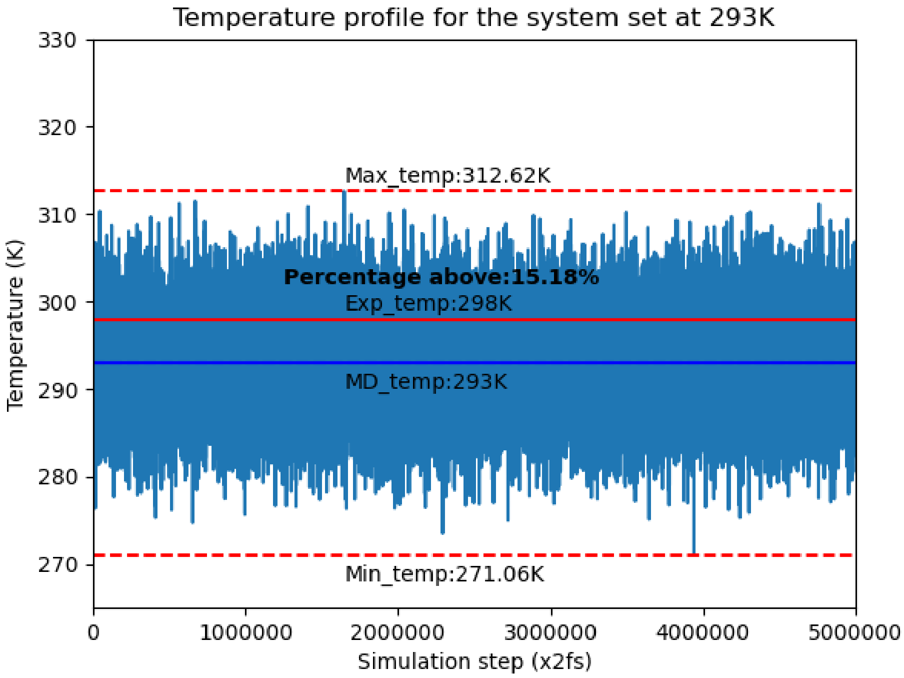

Figure S26: Temperature profile for the γ-CD systems set at 293K.

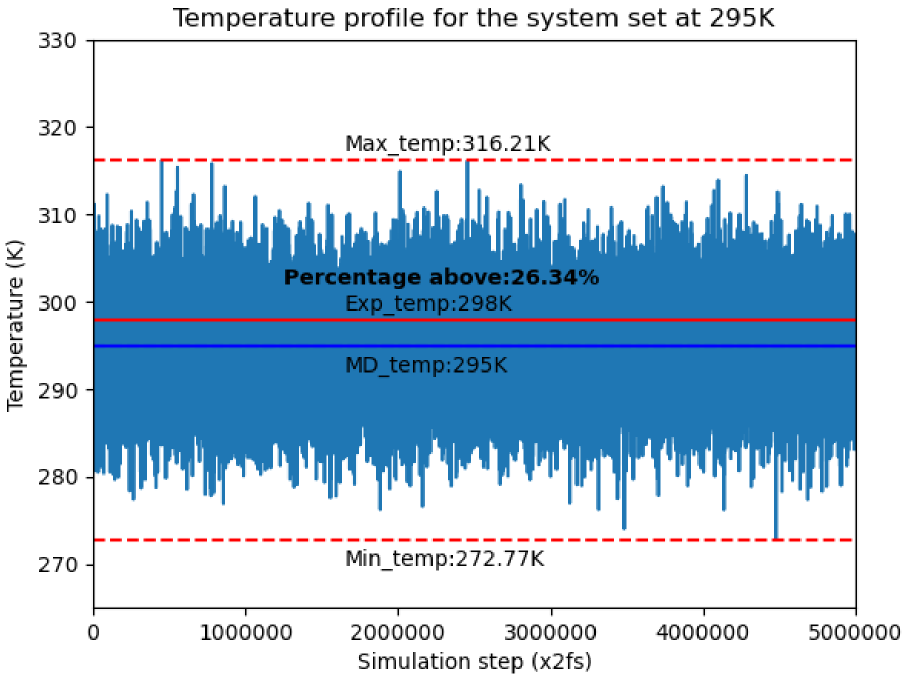

Figure S27: Temperature profile for the γ-CD systems set at 295K.

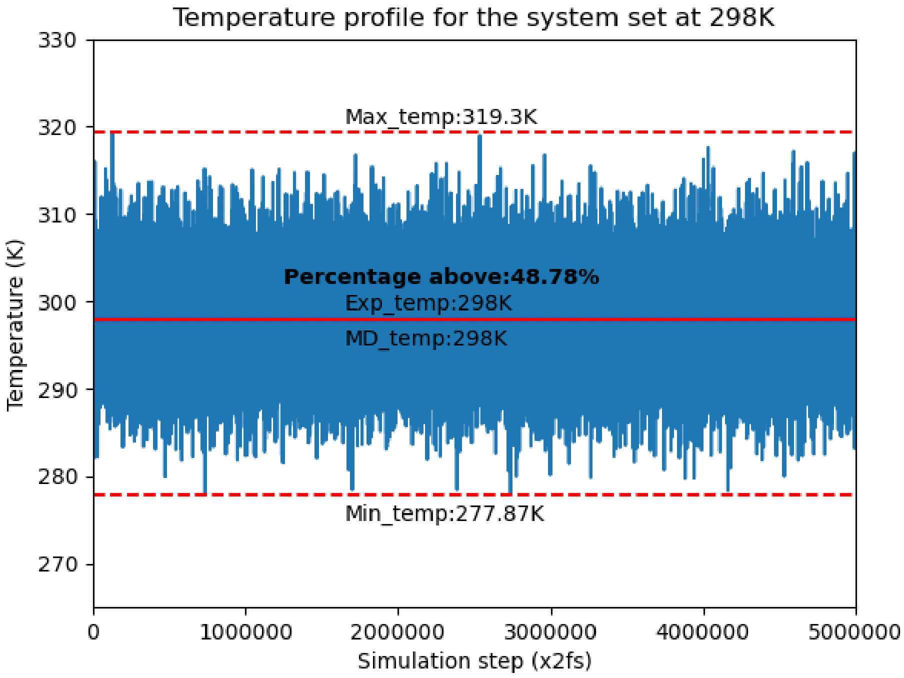

Figure S28: Temperature profile for the γ-CD systems set at 298K.

Table S1. Chemical shift for the RIF, γ-CD and ARG in free and complex forms.

^{1}H-NMR signals	**Pure components**	**Binary and multicomponent systems**		
		**RIF:ARG**	**RIF:γ-CD**	**RIF:γ-CD:ARG**
**RIF protons**	**δ RIF$_{free}$ (ppm)**	**δ RIF$_{complex}$ (ppm)**	**δ RIF$_{complex}$ (ppm)**	**δ RIF$_{complex}$ (ppm)**
H33	-0.2065	-0.2465	-0.2523	-0.2600
H31	0.6175	0.5910	0.5920	-0.5530
H32	0.9680	0.9390	0.9370	0.9010
H26	1.1240	1.2100	1.1935	1.1670
H24	1.4210	1.3850	1.3755	1.3565
H13	1.8250	1.7950	1.7610	1.6920
H22	1.8735	1.8910	1.8675	1.9580
H14	2.0500	2.0210	2.0310	2.0070
H30	2.0550	2.0420	2.0110	2.0110
H36	2.1220	2.0910	2.0860	2.0590
H20	2.3980	2.3725	2.3735	2.3285
H4'	2.6610	2.6970	2.7890	2.4140
H37	3.0640	3.0320	3.0305	3.0090
H3'-H5'	3.1115	3.1100	3.0895	3.0430
H2'-H6'	3.3140	3.2390	3.2340	3.2825
H27	3.4745	3.4450	3.4410	3.4020
H21	3.8440	3.8130	3.8500	3.8700
H28	5.1605	5.1430	5.1660	5.0810
H19	6.1875	6.1810	6.1810	6.1030
H29	6.3650	6.4555	6.3385	6.2930
H17	6.4780	6.4985	6.4655	6.4065
H18	6.8505	6.8540	6.9155	6.7675
H1'	8.0550	8.0370	8.0720	8.0010
**γ-CD protons**	**δ γ-CD$_{free}$ (ppm)**	**δ γ-CD$_{complex}$ (ppm)**	**δ γ-CD$_{complex}$ (ppm)**	**δ γ-CD$_{complex}$ (ppm)**
H4	3.5855	-	3.5875	3.5620
H2	3.6480	-	3.6465	3.6100
H5-H6	3.8660	-	3.8520	3.7900
H3	3.9310	-	3.9270	3.8950
H1	5.1055	-	5.0995	5.5085
**ARG protons**	**δ ARG$_{free}$ (ppm)**	**δ ARG$_{complex}$(ppm)**	**δ ARG$_{complex}$(ppm)**	**δ ARG$_{complex}$(ppm)**
H4-H3	1.7605	1.7950	-	1.8000
H5	3.2375	3.2390	-	3.1965
H2	3.4800	3.4550	-	3.4015
